# Molecular mechanism of prolactin-releasing peptide recognition and signaling via its G protein-coupled receptor

**DOI:** 10.1038/s41421-024-00724-6

**Published:** 2024-09-03

**Authors:** Yang Li, Qingning Yuan, Xinheng He, Yumu Zhang, Chongzhao You, Canrong Wu, Jingru Li, H. Eric Xu, Li-Hua Zhao

**Affiliations:** 1 State Key Laboratory of Drug Research, Center for Structure and Function of Drug Targets, Shanghai Institute of Materia Medica, Chinese Academy of Sciences, Shanghai, China; 2 University of Chinese Academy of Sciences, Beijing, China; 3 School of Chinese Materia Medica, Nanjing University of Chinese Medicine, Nanjing, Jiangsu China; 4 Translational Center for Medicinal Structural Biology, Ruijin Hospital, Shanghai Jiao Tong University School of Medicine, Shanghai, China

**Keywords:** Cryoelectron microscopy, Molecular biology

## Abstract

Prolactin-releasing peptide (PrRP) is an RF-amide neuropeptide that binds and activates its cognate G protein-coupled receptor, prolactin-releasing peptide receptor (PrRPR), also known as GPR10. PrRP and PrRPR are highly conserved across mammals and involved in regulating a range of physiological processes, including stress response, appetite regulation, pain modulation, cardiovascular function, and potentially reproductive functions. Here we present cryo-electron microscopy structures of PrRP-bound PrRPR coupled to G_q_ or G_i_ heterotrimer, unveiling distinct molecular determinants underlying the specific recognition of the ligand’s C-terminal RF-amide motif. We identify a conserved polar pocket that accommodates the C-terminal amide shared by RF-amide peptides. Structural comparison with neuropeptide Y receptors reveals both similarities and differences in engaging the essential RF/RY-amide motifs. Our findings demonstrate the general mechanism governing RF-amide motif recognition by PrRPR and RF-amide peptide receptors, and provide a foundation for elucidating activation mechanisms and developing selective drugs targeting this important peptide–receptor system.

## Introduction

Neuropeptides, with over 100 identified types, are the most abundant signaling molecules in the nervous system. Among these, RF-amide peptides, identifiable by their C-terminal Arg-Phe-NH_2_ (RF-amide) motif, play vital roles as neurotransmitters and neuromodulators. They are involved in a range of physiological processes, including metabolism, pain perception, and reproduction^1^. In mammals, the RF-amide peptide subfamily comprises prolactin-releasing peptide (PrRP), neuropeptide FF (NPFF), kisspeptin, RF-amide-related peptide (RFRP), and pyroglutamylated RF-amide peptide (QRFP). These neuropeptides interact with five G protein-coupled receptors (GPCRs): prolactin-releasing peptide receptor (PrRPR, GPR10)^2^, neuropeptide FF receptor 1/2 (NPFF1R/NPFF2R, GPR147/GPR74)^3,4^, kisspeptin receptor (KISS1R, GPR54)^5^, and pyroglutamylated RF-amide peptide receptor (QRFPR, GPR103)^6^. Notably, PrRP and PrRPR are highly conserved across mammals and are integral in regulating behaviors and physiological processes through the endocrine system.

**Fig. 1:**
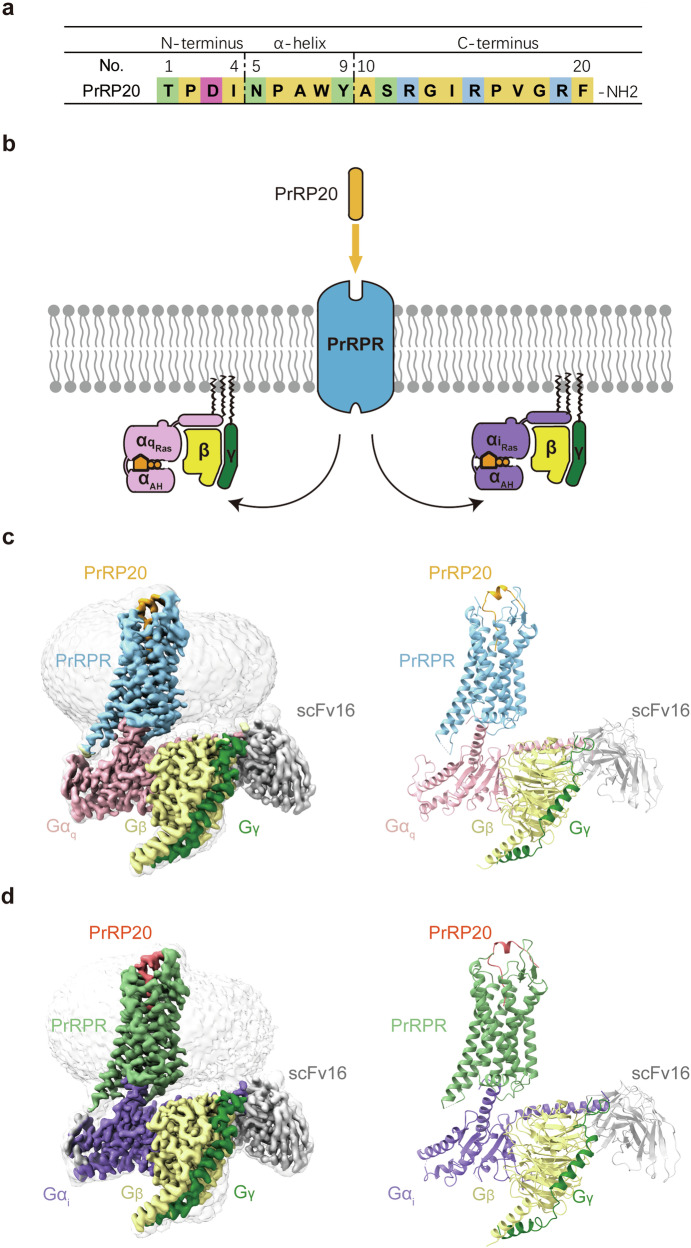
Overall structures of PrRP20–PrRPR–G_q_ and PrRP20–PrRPR–G_i_ complexes. **a** Sequence of PrRP20. Residues are shown in green, magenta, light blue, and yellow, which represent polar, acidic, basic, and hydrophobic amino acids, respectively. **b** Schematic illustration of G protein coupling of PrRPR activated by PrRP20. **c**, **d** Cryo-EM density maps (left panel) and cartoon representation (right panel) of PrRP20–PrRPR–G_q_–scFv16 (**c**) and PrRP20–PrRPR–G_i_–scFv16 (**d**) complexes. Components of PrRPR complexes are colored as indicated.

PrRP, first isolated from bovine hypothalamus, has two biologically active isoforms, PrRP31 and PrRP20. PrRP20 is a 20-amino acid peptide with amidation at its C-terminus (Fig. 1a)^2^. Identified as the endogenous ligand for GPR10 through reverse pharmacology, PrRP20 shows a high affinity to this receptor^2,7^. PrRPR, primarily triggering G_q/11_ signaling pathways, is also suggested to engage G_i/o_ pathways (Fig. 1b)^8^. PrRPR is abundantly expressed in the thalamic reticular nucleus, hypothalamic nuclei, area postrema, and nucleus of the solitary tract^9,10^. These regions regulate functions like stress, appetite, pain and reproduction. Though initially posited to govern prolactin release, prevailing evidence suggests that PrRP/PrRPR regulate food intake, energy metabolism^11,12^, pain perception^13^, stress response^14^, endocrine^15^, sleep and rhythm^16,17^, and confer neuroprotection^18–20^.

Structure-activity relationship studies on PrRP analogs, focusing on the C-terminal RF-amide motif, have been crucial for understanding the interaction between PrRP and PrRPR^2,9,21^. These studies suggest that key amino acids for this interaction are located at the ligand’s C-terminus, and alterations in this region can significantly impact PrRP’s activity. This finding has important implications for the development of therapeutic applications targeting obesity, type 2 diabetes, sleep disorders, and epilepsy^22,23^.

However, despite their therapeutic potential, the complex interaction network between RF-amide peptides and their receptors^24–26^, along with a lack of structural information, has hindered a comprehensive understanding of their mechanisms. This study aims to bridge this gap by reporting cryo-electron microscopy (cryo-EM) structures of PrRP20-bound PrRPR coupled to G_q_ and G_i_, respectively, shedding light on peptide agonist recognition and receptor activation. Our findings clarify the mechanism governing RF-amide motif recognition by PrRPR and provide a foundation for the rational design of selective drugs targeting this important peptide–receptor system.

## Results

### Cryo-EM analysis and overall structure

To facilitate the expression of these complexes, two maltose-binding protein (MBP) tags were introduced at the C-terminus of the wild-type (WT) full-length receptor, which also served as an affinity purification tag^27^. In addition, we employed the NanoBiT strategy to stabilize the GPCR–G protein complex, wherein the large subunit (LgBiT) and its complementary high-affinity peptide (HiBiT) (SmBiT or peptide 86) were fused to the C-terminus of the receptor and Gβ subunit, respectively^28,29^. Two types of Gα proteins were used to assemble the PrRPR complex: engineered Gα_q_ and Gα_i_^30^. Incubation of the endogenous ligand PrRP20 with membranes from cells co-expressing PrRPR and Gα_q_ or Gα_i_ heterotrimers in the presence of scFv16 enables efficient assembly of the PrRP20–PrRPR–Gα_q_ and PrRP20–PrRPR–Gα_i_ complexes, producing highly homogeneous complex samples for structural studies^31^.

The PrRP20–PrRPR–G_q_–scFv16 and PrRP20–PrRPR–G_i_–scFv16 complex structures were determined using single-particle cryo-EM, achieving global resolutions of 2.96 Å and 2.97 Å, respectively (Fig. 1c, d; Supplementary Figs. S1, S2 and Table S1). The EM maps enabled accurate model building of the receptor residues Q54^NTD^ to G259^5.72^ and A266^6.23^ to V351^8.59^ (superscripts indicate Ballesteros-Weinstein numbering) for the PrRP20–PrRPR–G_q_–scFv16 complex, and Q54^NTD^ to V256^5.69^ and A266^6.23^ to V351^8.59^ for the PrRP20–PrRPR–G_i_–scFv16 complex (Supplementary Fig. S3). These maps were also clear for most of the residues in the ligand and G_q_ or G_i_ heterotrimers, providing detailed information about the ligand-binding pocket and the receptor–G protein coupling interface (Fig. 1c, d; Supplementary Fig. S3).

**Fig. 2:**
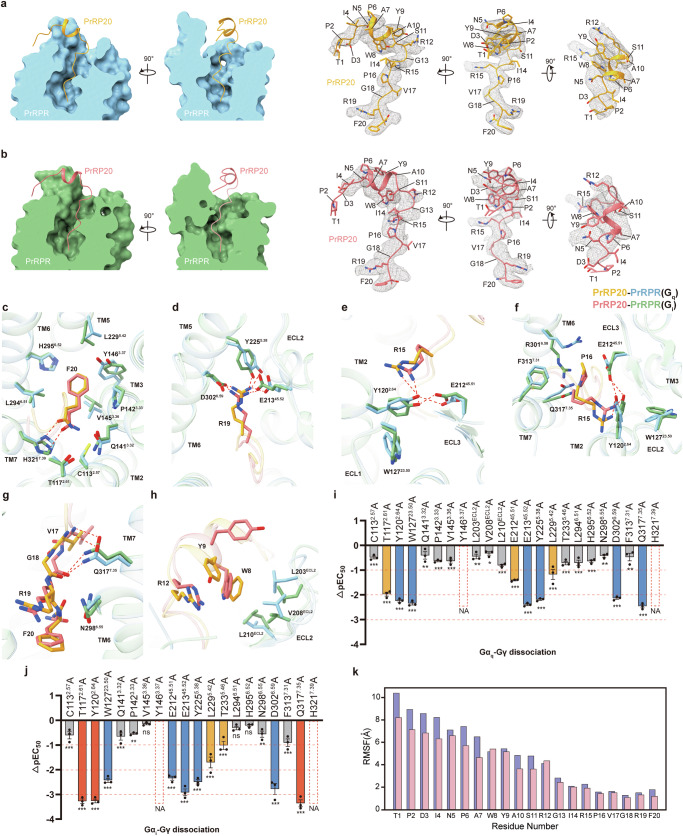
Recognition of PrRP20 by PrRPR. **a**, **b** Cross-section of the PrRP20-binding pocket in G_q_-coupled PrRPR (**a**) and G_i_-coupled PrRPR (**b**). The three-view drawing of density maps of PrRP20 are shown as grey meshes, and PrRP20 are displayed as orange (G_q_-coupled) or pink (G_i_-coupled) cartoons and sticks. **c**–**h** Detailed interactions of PrRP20 with residues in PrRPR. The binding sites of F20 (**c**), R19 (**d**), R15 (**e**), P16 and R15 (**f**), backbone of F20–V17 (**g**), R12, Y9 and W8 (**h**) are shown. Hydrogen bonds and salt bridges are depicted as red dashed lines. PrRP20 molecules are shown as cartoons and sticks. PrRPR is shown in blue (G_q_-coupled) or green (G_i_-coupled). **i**, **j** BRET2 assay to evaluate PrRP20-induced dissociation of heterotrimeric G_q_ protein (**i**) and G_i_ protein (**j**) (ΔpEC_50_ = pEC_50_ of PrRP20 to a specific PrRPR variant – pEC_50_ of PrRP20 to WT PrRPR; yellow column means ΔpEC_50_ ≤ –1; blue column means ΔpEC_50_ ≤ –2; red column means ΔpEC_50_ ≤ –3). Data are presented as means ± SEM; *n* = 3 independent samples, each consisting of triplicate measurements. Significance was determined by one-way ANOVA with Dunnett’s multiple comparisons test. NA, no activity; ns, not significant; *P < 0.05; **P < 0.01; ***P < 0.001. Exact *P* values are provided in Supplementary Table S2. **k** Ligand RMSF of each residue in G_q_-coupled (pink) and G_i_-coupled (purple) PrRPR complexes.

PrRPR adopts a seven-transmembrane folding conformation typical for G protein recruitment, and PrRP20 presents an L-shaped conformation (Fig. 2a, b). The two complex structures are highly similar, with root mean square deviation (RMSD) values of 0.501 Å for the complex and 0.581 Å for the receptor. In both structures, the EM maps showed clarity for PrRP20, except for the N-terminal 3 amino acids, with its C-terminus inserted into the ligand-binding pocket, consistent with the previous structure-activity relationship studies^2,9,21^. Unlike the previously reported α-helical structure for the C-terminal residues A10–G13 and R15–R19 of PrRP20^32,33^, these residues instead adopted a more relaxed, curled conformation in our structure (Fig. 2a, b). In the activated-state structure of the neuropeptide Y (NPY) receptor with the highest homology to PrRPR, the C-terminal region of its ligand NPY exhibited similar conformational changes^34,35^, indicating a common activation conformation for the two neuropeptides. Furthermore, an unexpected α-helical structure was found in the originally flexible N-terminal residues N5–Y9 of PrRP20, corresponding to the chemical environment of the sub-pocket at the receptor’s top.

### Binding modes of PrRP20 for PrRPR

In the PrRP20–PrRPR–G_q_–scFv16 and PrRP20–PrRPR–G_i_–scFv16 structures, PrRP20 assumes a similar conformation within the ligand-binding pocket, defined by extracellular loops (ECLs) and transmembrane helices TM2, TM3, and TM5–TM7 of PrRPR (Fig. 2a, b). PrRP20 is organized into three distinct segments: the N-terminus (T1–I4), an α-helix (N5–Y9), and the extended C-terminus (A10–F20) (Fig. 1a). The C-terminus, forming an L-shaped arrangement with the other segments, inserts into the transmembrane helical bundle, crucial for receptor activation. At the bend located at R12, PrRP20 aligns its N-terminus and α-helix nearly parallel to the receptor’s extracellular surface. The α-helix interacts with ECL2, while the N-terminus faces the extracellular tip of TM4 (Fig. 2a, b).

At the base of the ligand-binding pocket, the conserved residues of RF-amide peptides, R19 and F20, create a sub-pocket (Fig. 2a, b). Here, the amide group of F20 engages in a polar network with C113^2.57^, T117^2.61^, Q141^3.32^, and H321^7.39^, indicating its importance in PrRP20’s affinity (Fig. 2c). The acidic nature of this network repels the negatively charged unacetylated C-terminus of PrRP20^2,9^. On the opposite side, the hydrophobic pocket surrounding the side chain of F20 includes P142^3.33^, V145^3.36^, Y146^3.37^, L229^5.42^, L294^6.51^, and H295^6.52^ from TM3, TM5, and TM6, as confirmed by alanine mutagenesis analysis (Fig. 2c, i, j; Supplementary Table S2). In addition, R19 forms polar interactions with D302^6.59^, Y225^5.38^, and E213^45.52^. These interactions are crucial for peptide recognition (Fig. 2d), as indicated by a drastic reduction in PrRP20 potency upon mutation of these residues (Fig. 2i, j). Accordingly, our observations agree with the previous studies that demonstrated the importance of R19 and the acylated and bulky side chain of F20 for receptor activation^9,21,36^.

The interaction between PrRP20 and the upper sub-pocket of PrRPR, near the receptor’s extracellular surface, includes residues P16–W8 from PrRP20. In this interaction network, R15 is particularly essential^9^, as it interacts with receptor Y120^2.64^ (Fig. 2e). Y120^2.64^’s dual interaction with E212^45.51^ and π–π stacking with W127^23.50^ stabilizes the folding of the extracellular region (Fig. 2e). Mutations in these residues significantly reduce ligand efficacy in activating both G_q_ and G_i_ proteins (Fig. 2i, j). The upper sub-pocket is sealed off by residues including Y120^2.64^, E212^45.51^ and those surrounding P16 (R301^6.58^, F313^7.31^, and Q317^7.35^), with Q317^7.35^ anchoring PrRP20’s backbone through polar interactions (Fig. 2f, g). The Q317^7.35^A mutation most significantly reduced the potency of PrRP20-mediated G_q_ and G_i_ activation (Fig. 2i, j).

Deletion analysis of PrRP20’s N-terminal region revealed that the shortest effective analog is a C-terminal heptapeptide (I14–F20), but with substantially lower affinity^9^. This suggests that the α-helix and N-terminal region of PrRP20 may also contribute to receptor binding. In both structures, the intramolecular motif of W8, Y9, and R12 within PrRP20 stabilizes its α-helical structure (Fig. 2h). Additionally, hydrophobic interactions between W8 and the receptor’s ECL2 (L203^ECL2^, V208^ECL2^, L210^ECL2^) are pivotal, as alanine mutations in these residues diminish ligand efficacy (Fig. 2i). This finding aligns with the structure-activity relationship studies of the PrRP20 analog (W8–F20), which did not show reduced activity in vitro^21^.

We have developed molecular dynamics (MD) simulation systems for ligand–PrRPR–G_q_ and –G_i_ complexes, conducting three parallel simulations of 200 ns each to examine the dynamics. Initially, we assessed biased signaling through the estimation of binding free energies using MMGBSA. The binding free energies for G_q_ and G_i_ were calculated as –51.93 ± 6.99 kcal/mol and –44.15 ± 2.96 kcal/mol, respectively. These results support the notion of biased signaling towards G_q_, demonstrating that the simulation trajectories are consistent with experimental findings. Further analysis involved calculating the root-mean-square fluctuation (RMSF) of all heavy atoms in the ligand for each residue, as depicted in Fig. 2k. The analysis revealed that G_i_ binding results in a more flexible ligand, particularly at residues T1, P2, and D3, consistent with the observed density loss. Notably, the regions beyond residue G13 displayed greater stability compared to the external segments (Fig. 2k).

Sequence alignment of PrRP across species shows the high conservation of the C-terminal tridecapeptide, especially in crucial ligand–receptor interaction residues, except for V17 (Supplementary Fig. S6a). This can be explained as V17 interacts with the receptor through its main chain peptide bond (Fig. 2g). Correspondingly, receptor residues interacting with this tridecapeptide are also highly conserved (Supplementary Fig. S6b, c), underscoring a conserved evolutionary mechanism for PrRP–PrRPR interaction across species.

### Comparative analysis of the recognition mechanisms for RF/RY-amide motifs in PrRPR and other receptors

Our analysis expands to encompass other RF-amide peptides and the similar RY-amide peptides, examining their C-terminal motif interactions with respective receptors. PrRP20, part of the RF-amide family alongside NPFF, Kisspeptin, RFRP, and QRFP, shares the highly conserved C-terminal RF-amide (Supplementary Fig. S7a). We compared the amino acid residues of RF-amide peptide receptors (PrRPR, NPFF1R, NPFF2R, KISS1R, and QRFPR) to determine a common recognition mechanism for this motif (Supplementary Fig. S7b, c). Except for the substitution of H^7.39^ by the similarly polar residue Q^7.39^ in QRFPR, the residues C^2.57^, T^2.61^, Q^3.32^, and H^7.39^, key to the C-terminal amide recognition in PrRP20, are largely conserved, indicating a shared polar interaction network. However, variations exist around R19, with two hydrophobic residues mutated in KISS1R, but E^45.52^, Y^5.38^, and D/E^6.59^ remain conserved. The conservation around F20’s side chain is less stringent, but a general hydrophobic pocket was observed for all receptors, suggesting a common mechanism for recognizing the RF-amide motif. Therefore, our understanding of the interaction of PrRPR with the RF-amide may be applicable to other RF-amide peptide receptors.

**Fig. 3:**
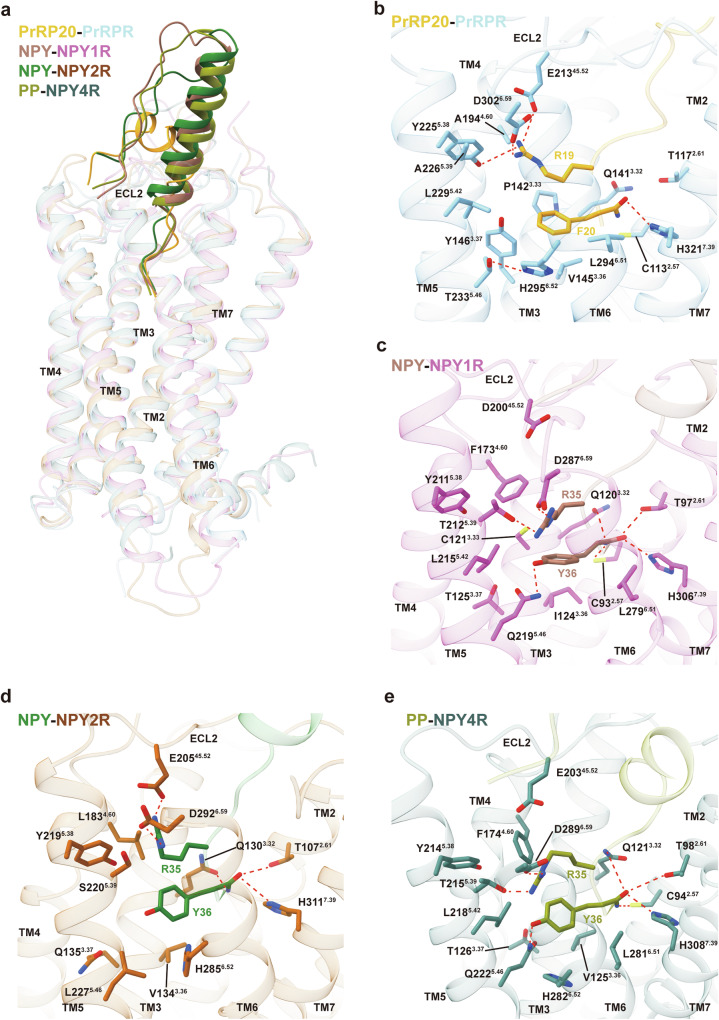
Structural comparison of RF-amide motif and RY-amide motif interactions in PrRP20–PrRPR and NPY/PP–NPYRs complexes. **a** Superimposition of structures of the PrRP20–PrRPR, NPY–NPY1R (PDB: 7X9A), NPY–NPY2R (PDB: 7X9B) and PP–NPY4R (PDB: 7X9C) complexes. **b**–**e** Binding mode of the RF-amide motif within the TMs of PrRPR (**b**) and RY-amide motif within the TMs of NPY1R (**c**), NPY2R (**d**), and NPY4R (**e**). Hydrogen bonds and salt bridges are depicted as red dashed lines.

The sequence homology between PrRPR and NPY receptors, specifically NPY1R, NPY2R, and NPY4R (Supplementary Fig. S8b), prompted a comparison between PrRP20 with their ligands, NPY and pancreatic polypeptide (PP), which possess a C-terminal RY-amide motif (Supplementary Fig. S8a). Structural comparisons of PrRP20–PrRPR complex with NPY–NPY1R, NPY–NPY2R, and PP–NPY4R complexes showed a high overlap in the binding posture of their ligands’ C-terminal R-F/Y residues (Fig. 3a). Sequence alignment indicates a certain level of conservation in the recognition patterns of NPYRs for the RY-amide motif, which are similar to the recognition mode of PrPRP for the RF-amide motif (Supplementary Fig. S8c).

Key residues like C^2.57^, T^2.61^, Q^3.32^, and H^7.39^, crucial for the activity of PrRP20 and NPY/PP, are conserved in both PrRPR and NPYRs, forming a polar network around the amide groups of F20 and Y36 (Fig. 3b–e; Supplementary Fig. S8c). However, differences emerge in the microenvironment around R19 and R35. While E^45.52^, Y^5.38^, and D^6.59^ remain highly conserved (Supplementary Fig. S8c), we observed that among NPYRs, only D^6.59^ forms a conserved polar interaction with R35 of NPY/PP (Fig. 3c–e). Notably, these interactions are disrupted by two key mutations in NPYRs: F/L^4.60^ and T^5.39^. The F/L^4.60^ mutation creates a steric clash with R35, pushing it away towards TM6. The T^5.39^ mutation enables R35 to form a new polar interaction with TM5 in NPY1R and NPY4R (Fig. 3c, e). Nevertheless, owing to the lesser steric hindrance of L^4.60^ in NPY2R, E205^45.52^ maintains its polar interaction with R35 (Fig. 3d).

The binding pockets for F20 of PrRP20 and Y36 of NPY/PP, though analogous, display different characteristics. In NPY1R and NPY4R, Y36’s hydroxyl group forms a polar interaction with Q^5.46^, critical for activity (Fig. 3c, e). However, this interaction is absent in NPY2R due to the L227^5.46^ mutation (Fig. 3d). In PrRPR, F20 lacks a hydroxyl group, preventing a similar interaction with T233^5.46^ (Fig. 3b). Interestingly, T233^5.46^ establishes a polar interaction with H295^6.52^, a feature absent in NPYRs. Additionally, Y^3.37^ in PrRPR, compared to T/Q^3.37^ in NPYRs, creates a more confined binding pocket for F20.

N^6.55^, highly conserved across PrRPR and NPYRs (Supplementary Fig. S8c), engages in different interactions. In PrRPR, it forms a polar interaction with the peptide bond between R19 and F20 of PrRP20 (Fig. 2g; Supplementary Fig. S9), while in NPYRs, it interacts with R33 of NPY/PP (Supplementary Fig. S9). At the equivalent position, the residue in PrRP20 is V17, which similarly forms an anchoring interaction with Q317^7.35^ in PrRPR (Fig. 2g). These discrepancies are due to sequence variations between PrRP20 and NPY/PP, excluding their RF/RY-amide motifs.

### Activation mechanism of PrRPR

**Fig. 4:**
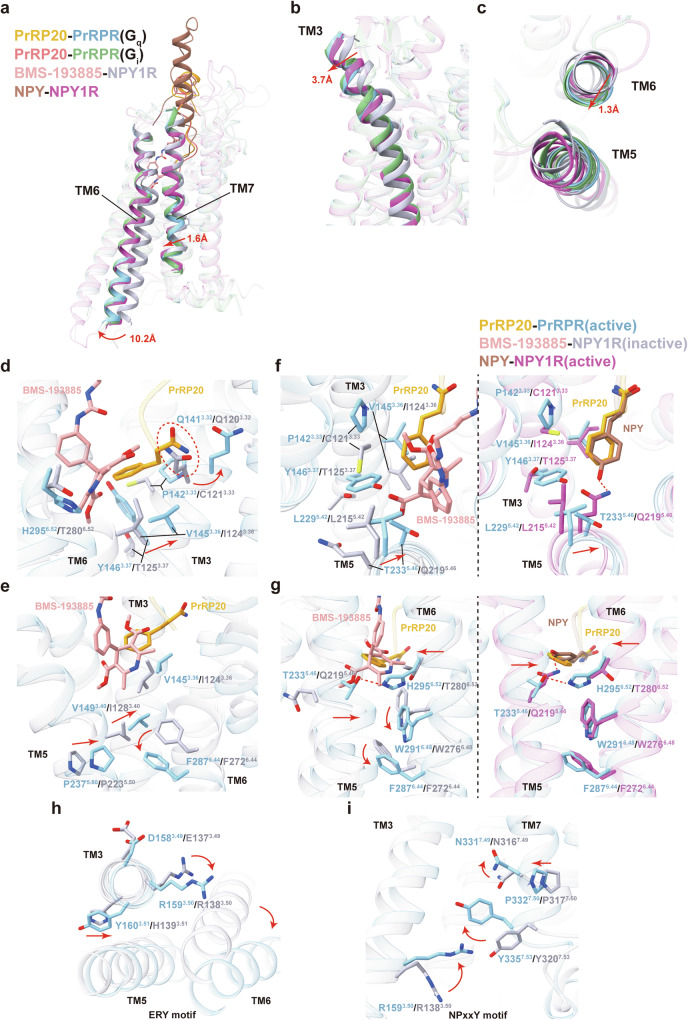
Activation mechanism of PrRPR. **a**–**c** Structure superposition of PrRPR–G_q_ and PrRPR–G_i_ complexes with the agonist (NPY)-bound NPY1R (PDB: 7X9A) and antagonist (BMS-193885)-bound NPY1R (PDB: 5ZBH). Side-view of the movement of TM6 and TM7 (**a**), TM3 (**b**) and top-view of the movement of TM6 (**c**) are shown. The movement directions of TMs in PrRPR relative to the inactive NPY1R are highlighted as red arrows. **d**–**i** Detailed interaction changes upon activation of PrRPR. Hydrogen bonds and salt bridges are depicted as red dashed lines. The movement orientations of amino acids and TMs are indicated as red arrows. Interactions between PrRP20 and residues located at the bottom of peptide-binding pocket (**d**). The possible clash is highlighted by a red dashed circle. The rearrangement of the residues in the PIF motif (**e**). The conformation comparison of PrRPR with antagonist-bound NPY1R (left panel) and PrRPR with agonist-bound NPY1R (right panel) (**f**, **g**). Conformational changes of the conserved “micro-switches” upon receptor activation, including ERY (**h**) and NPxxY (**i**) motifs.

To analyze the activation mechanism, we performed structural comparisons of the PrRP20–PrRPR complexes with the antagonist-bound NPY1R, which affirms that PrRPR is in an activated state in both structures. This activation is highlighted by the outward displacement at the cytoplasmic end of TM6 (10.2 Å, measured at the Cα atom of D269^6.26^/R254^6.26^) and an inward movement at the cytoplasmic end of TM7 (1.6 Å, measured at the Cα atom of F333^7.51^/I318^7.51^), typical hallmarks of class A GPCR activation (Fig. 4a). Unlike the inactive conformation of NPY1R, PrRPR exhibits an outward shift at the extracellular end of TM3 (3.7 Å, measured at the Cα atom of F138^3.29^/P117^3.29^) and a lateral shift of TM6 towards TM5 (1.3 Å, measured at the Cα atom of D302^6.59^/D287^6.59^) (Fig. 4b, c). These structural changes are similar yet more pronounced than those in the activated NPY–NPY1R–G_i_ complex. Owing to the similarity of the conformations in both PrRP20–PrRPR complexes, we focus on the PrRP20–PrRPR–G_q_ complex to elucidate PrRPR’s activation mechanism.

At the base of the ligand-binding pocket, a hydrophobic lock consisting of V145^3.36^, Y146^3.37^, and H295^6.52^ prevents direct contact between PrRP20’s C-terminal residue F20 and the “Toggle Switch” residue W291^6.48^ (Fig. 4d). The interactions between F20 and TM3 residues in PrRPR, particularly the polar interaction of its amide group with Q141^3.32^, trigger an upward rotational change in Q141^3.32^, which initiate PrRPR activation. F20’s phenylmethyl group also forms extensive hydrophobic interactions with P142^3.33^, Y146^3.37^, and V145^3.36^ (Fig. 4d), causing an outward shift of TM3’s extracellular side and rearranging the side chains of the PIF motif (V149^3.40^, P237^5.50^, F287^6.44^) (Fig. 4e). This mechanism mirrors NPY1R’s primary activation pathway^37^.

Notably, we additionally revealed a unique activation pathway initiated by Y146^3.37^ in PrRPR. The substitution of NPY1R’s residues C121^3.33^ and T125^3.37^ by P142^3.33^ and Y146^3.37^ in PrRPR results in a larger displacement of TM3’s extracellular side. This shift facilitates an interaction between Y146^3.37^ with L229^5.42^ and T233^5.46^ on TM5 (Fig. 4f). Alanine substitution analysis of these amino acids, especially Y146^3.37^, confirms their crucial role in PrRPR activation (Fig. 2i, j). In contrast, the shorter side chain of T125^3.37^ in NPY1R is unable to interact with L215^5.42^ and Q219^5.46^, and Q219^5.46^ is firmly anchored within the helical bundle due to polar interactions with Y36 (Fig. 4f). The steric hindrance between TM3 and TM5 residues in PrRPR prompts TM5 to move horizontally towards TM6, allowing T233^5.46^ to interact with H295^6.52^ and induce a counter movement of TM6, which cannot be induced by Q219^5.46^ and T280^6.52^ in NPY1R (Fig. 4g). Moreover, the movement of H295^6.52^ may prompt conformational changes in W291^6.48^, leading to a significant outward displacement of TM6’s cytoplasmic end. Given the specificity of Y^3.37^ (Supplementary Figs. S7c and S8c), we propose that this activation mechanism is distinctive from those of the other RF/RY-amide peptide receptors.

Furthermore, other conserved residues in “micro-switches” (ERY and NPxxY motifs) in PrRPR undergo conformational changes, similar to those in the active-like state of NPY1R relative to the antagonist-bound NPY1R (Fig. 4h, i). These changes transmit the activation signal from PrRP20 to PrRPR’s cytoplasmic side for G protein coupling. Together, these structural insights reveal the intricate mechanism of PrRPR activation, emphasizing the nuanced differences and similarities in GPCR activation pathways.

### PrRPR–G_q_/G_i_ coupling mechanism

**Fig. 5:**
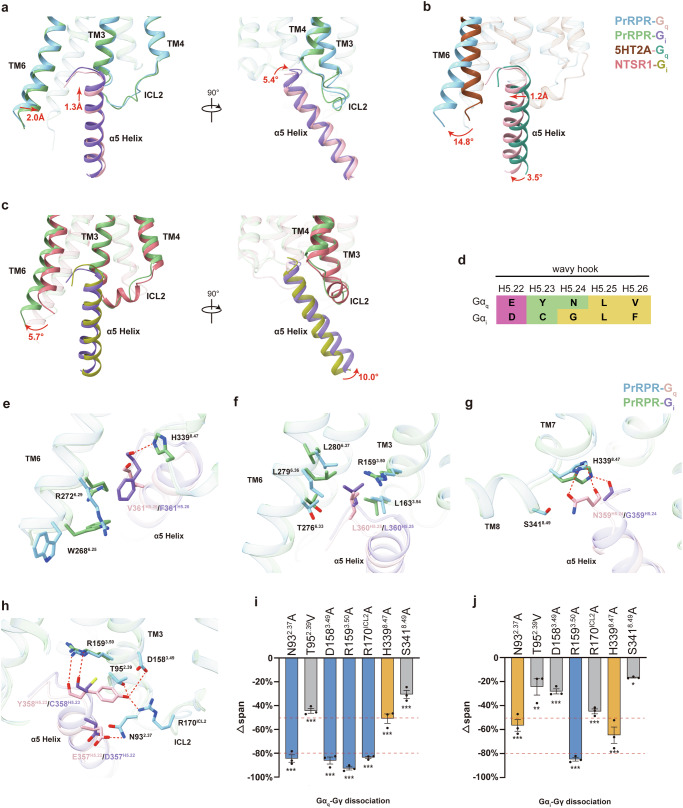
G_q_/G_i_ coupling of PrRPR. **a** An overall conformational comparison of G_q_-coupled and G_i_-coupled PrRPR. TM6, TM3, TM4 and ICL2 of receptors, as well as α5 helix of G proteins, are highlighted. **b** The conformational comparison of G_q_-coupled PrRPR with G_q_-coupled 5HT2A (PDB: 6WHA). **c** The conformational comparison of G_i_-coupled PrRPR with G_i_-coupled NTSR1 (PDB: 6OS9). **d** Alignment of the “wavy hook” in the extreme C-terminus (V/F^H5.26^–E/D^H5.22^) of Gα_q_ and Gα_i_. Residues are shown in green, magenta, and yellow, which represent polar, acidic, and hydrophobic amino acids, respectively. **e**–**h** Distinct interaction patterns of residues from the “wavy hook” motif. Details of the interaction between PrRPR and V/F^H5.26^ (**e**), L^H5.25^ (**f**), N/G^H5.24^ (**g**), Y/C^H5.23^ and E/D^H5.22^ (**h**) of G_q_ and G_i_ subunits. Hydrogen bonds and salt bridges are depicted as red dashed lines. **i**, **j** BRET2 assay to evaluate PrRP20-induced dissociation of heterotrimeric G_q_ protein (**i**) and G_i_ protein (**j**) (Δspan = the span value of PrRP20 to a specific PrRPR variant – the span value of PrRP20 to WT PrRPR; yellow column means Δspan ≤ –50%; blue column means Δspan ≤ –80%). Data are presented as means ± SEM; *n* = 3 independent samples, each consisting of triplicate measurements; significance was determined by one-way ANOVA with Dunnett’s multiple comparisons test. *P < 0.05; **P < 0.01; ***P < 0.001. Exact span values are provided in Supplementary Table S3.

Our analysis reveals notable distinctions in the PrRPR–G protein complex structures, particularly in the conformations of TM6 and the α5 helix of the Gα subunit. In the PrRPR–G_i_ complex, TM6 exhibits an inward shift compared to PrRPR–G_q_ complex (2.0 Å, measured at the Cα atom of W268^6.25^), necessitating an adjustment of the α5 helix of the Gα_i_ subunit into the transmembrane helical bundle (1.3 Å, measured at the Cα atom of E357/D357^H5.22^, where superscripts refer to the common Gα numbering system^38^) and towards intracellular loop 2 (ICL2, 5.4°) to avoid clashes (Fig. 5a). This adjustment is in contrast with other G protein-coupled class A GPCRs. For instance, TM6 in PrRPR–G_q_ complex shows a more significant outward shift (14.8°) compared to the 5HT2A–G_q_ complex, influencing the α5 helix orientation with a displacement of 1.2 Å (measured at the Cα atom of E357^H5.22^) at the C-terminus and a rotation of 3.5° at the N-terminus (Fig. 5b). Similarly, in PrRPR–G_i_ complex, an outward displacement (5.7°) of TM6 relative to NTSR1–G_i_ complex is accompanied by a tilt (10.0°) of the α5 helix towards ICL2 (Fig. 5c).

The interaction interface between the receptor cytoplasmic cavity and the extreme C-terminal “wavy hook” of the α5 helix (Fig. 5d), which thought to be one of the critical determinants of G protein selection^39,40^, varies between the PrRPR–G_q_ and PrRPR–G_i_ complexes. In the PrRPR–G_i_ structure, F361^H5.26^ faces spatial constraints from residues W268^6.25^ and R272^6.29^ of TM6 with its carbonyl interacting with H339^8.47^ in helix 8, while in the PrRPR–G_q_ structure, a more relaxed conformation exists due to the smaller V361^H5.26^ side chain and the rotated W268^6.25^ and R272^6.29^ (Fig. 5e). These conformational differences are attributed to the environmental variations surrounding V/F^H5.26^. However, L360^H5.25^, highly conserved across G protein families, occupies a similar hydrophobic pocket in both complexes, interacting with residues in TM3 and TM6 (Fig. 5f).

Additional contacts are observed between N359^H5.24^ of Gα_q_ and H339^8.47^ and S341^8.49^ of PrRPR in the PrRPR–G_q_ complex, while only the interaction between the carbonyl of G359^H5.24^ in Gα_i_ and H339^8.47^ of PrRPR is retained in the PrRPR–G_i_ complex (Fig. 5g). Similarly, the carbonyl groups of Y358^H5.23^ in PrRPR–G_q_ and C358^H5.23^ in PrRPR–G_i_ both interact with R159^3.50^. H339^8.47^A and R159^3.50^A mutations at the PrRPR–G protein interface significantly reduced the efficacy in activating both the G_q_ and G_i_ pathways (Fig. 5i, j). However, Y358^H5.23^ extends to TM2, TM3, and ICL2, forming a polar network with T95^2.39^, D158^3.49^, and R170^ICL2^, which is absent in the case of C358^H5.23^ (Fig. 5h). In addition, only E357^H5.22^ in Gα_q_ engages in a polar interaction with N93^2.37^ of PrRPR, while D357^H5.22^ in Gα_i_ does not (Fig. 5h), suggesting more extensive interactions between the receptor and G_q_ in the “wavy hook” region. Mutations of these residues in the receptor had a greater impact on G_q_ activation efficacy compared to G_i_ activation (Fig. 5i, j). These findings highlight the importance of the “wavy hook” in the selective coupling of PrRPR to G protein subtypes, particularly in its preference for G_q_ over G_i_ activation.

## Discussion

PrRPR, the exclusive receptor in the PrRPR family, has no published crystal or cryo-EM structures to date. Our cryo-EM structures of PrRPR bound to PrRP20, coupled with heterotrimeric G_q_ and G_i_ proteins, respectively, provide an in-depth view of ligand recognition, receptor activation, and G protein coupling.

The ligand-binding studies highlight the critical role of the C-terminal RF-amide motif in PrRP20. Our findings show that this motif, especially the conserved residues R19 and F20, forms a unique polar and hydrophobic interaction network within PrRPR, crucial for ligand affinity and specificity. The conservation of this motif across different RF-amide peptide receptors suggests a universal mechanism for RF-amide recognition. The observed differences in the binding of the RY-amide motif between PrRPR and NPYRs provide structural insights into their selective ligand binding and functional diversity.

Our analysis of PrRPR’s activation mechanism reveals significant conformational changes in TM3, TM5, and TM6. The pivotal role of F20 in initiating these changes, particularly its interaction with Q141^3.32^ and Y146^3.37^, underscores a unique activation pathway in PrRPR, distinct from other class A GPCRs. The comparison with NPY1R highlights the receptor-specific nuances in GPCR activation.

The PrRPR–G_q_/G_i_ coupling mechanisms, as revealed by our structures, underscore the importance of the Gα subunit’s α5 helix in determining G protein coupling specificity. The “wavy hook” region plays a critical role in the selective coupling of PrRPR to G_q_ and G_i_. This selective coupling is crucial for the downstream signaling pathways mediated by PrRPR.

These findings have broad implications for our understanding of GPCR function and drug development. The detailed mechanism of ligand recognition and receptor activation could aid in the design of novel therapeutics targeting PrRPR-related physiological processes, including appetite regulation, stress response, and pain modulation. Moreover, our study contributes to the broader field of GPCR research by providing a structural template for understanding the interaction networks between RF-amide peptides and their receptors.

## Materials and methods

### Constructs

The full-length human PrRPR was cloned into the pFastBac (Thermo Fisher Scientific) vectors using the ClonExpress II One Step Cloning Kit (Vazyme Biotech), along with the N-terminal haemagglutinin (HA) signal peptide. LgBiT was added at the end of helix 8 with a 15-amino acid polypeptide linker in between, followed by a TEV protease cleavage site and an OMBP-MBP tag^27^. The engineered Gα_q_ and Gα_i_ chimeric proteins were designed as chimeras based on the mini-Gα_s/q_71 and mini-G_s/i1_43 skeletons^30^, respectively, with the N-terminal 1–18 amino acids and the GαAH domain of G_i1_ replaced to facilitate binding to scFv16^30,41–43^. Human WT Gβ1, human Gγ2, and scFv16, as well as a Gβ1 fused with SmBiT at its C-terminus, were cloned into pFastBac vectors.

### Insect cell expression

Human PrRPR, G_q_ chimera, G_i_ chimera, Gβ1, Gγ, and scFv16 were co-expressed in Sf9 insect cells using the baculovirus method (Expression Systems). Cell cultures were grown in ESF 921 serum-free medium (Expression Systems) to a density of 3 million cells per mL and then infected with six separate baculoviruses at a suitable ratio. The culture was collected by centrifugation 48 h after infection, and cell pellets were stored at −80 °C.

### Complex purification

Cell pellets were thawed in 20 mM HEPES, pH 7.4, 100 mM NaCl, 10 mM MgCl_2_, and CaCl_2_ supplemented with Protease Inhibitor Cocktail (TargetMol). For the PrRP20–PrRPR–G_q_–scFv16 and PrRP20–PrRPR–G_i_–scFv16 complexes, 10 μM PrRP20 (TGpeptide) and 25 mU/mL apyrase (Sigma) were added. The suspension was incubated for 1 h at room temperature, and the complex was solubilized from the membrane using 0.5% (w/v) lauryl maltose neopentylglycol (LMNG) (Anatrace) and 0.1% (w/v) cholesteryl hemisuccinate (CHS) (Anatrace) for 2 h at 4 °C. Insoluble material was removed by centrifugation at 70,000× *g* for 35 min, and the supernatant was purified by MBP affinity chromatography (Dextrin Beads 6FF, SMART Lifesciences). The resin was then packed and washed with 30 column volumes of 20 mM HEPES, pH 7.4, 100 mM NaCl, 0.01% (w/v) LMNG, 0.002% CHS, and 2 μM ligand. The complex sample was eluted in buffer containing 20 mM HEPES, pH 7.4, 100 mM NaCl, 0.01% (w/v) LMNG, 0.002% CHS, 2 μM ligand, and 10 mM maltose. Complex fractions were concentrated with a 100-kDa molecular weight cut-off (MWCO) Millipore concentrator for further purification. The complex was then subjected to size-exclusion chromatography on a Superdex 6 Increase 10/300 GL column (GE Healthcare) pre-equilibrated with size buffer containing 20 mM HEPES, pH 7.4, 100 mM NaCl, 0.00075% (w/v) LMNG, 0.00025% (w/v) GDN (Anatrace), 0.0002% CHS, and 2 μM ligand to separate complexes. Eluted fractions were evaluated by SDS-PAGE, and those consisting of receptor–G_q_ and receptor–G_i_ protein complexes were pooled and concentrated for cryo-EM experiments.

### Cryo-EM grid preparation and data acquisition

Three microliters of the purified complexes at ~3 mg/mL and 11 mg/mL for PrRP20–PrRPR–G_q_ and PrRP20–PrRPR–G_i_ complexes, respectively, were applied onto a glow-discharged Quantifoil R1.2/1.3 300-mesh gold holy carbon grid. The sample was incubated for 5 s on the grids before blotting for 3 s under 100% humidity at 4 °C and then vitrified by plunging into liquid ethane using a Vitrobot Mark IV (Thermo Fisher Scientific). For both PrRPR complexes, cryo-EM data collection was performed on a Titan Krios G4 at a 300 kV accelerating voltage at the Advanced Center for Electron Microscopy, Shanghai Institute of Material Medica.

A total of 10,501 movies were collected for the PrRP20–PrRPR–G_q_ complex using a Falcon 4 detector in EER mode with a pixel size of 0.81 Å using the EPU. Movies were obtained at a dose rate of ~8 electrons per Å^2^ per second with a defocus ranging from –0.8 μm to −1.8 μm.

For PrRP20–PrRPR–G_i_ complex, a total of 7129 movies were collected by a Gatan K3 detector at a pixel size of 0.824 Å using the EPU. The micrographs were recorded in super-resolution mode at a dose rate of ~15 electrons per Å^2^ per second with a defocus ranging from −0.8 μm to −1.8 μm. The total exposure time was 2.35 s, and 36 frames were recorded per micrograph.

### Cryo-EM data processing

MotionCor2 was used to perform the frame-based motion-correction algorithm to generate a drift-corrected micrograph for further processing, and CTFFIND4 provided the estimation of the contrast transfer function (CTF) parameters in CryoSPARC^44,45^.

For the PrRP20–PrRPR–G_q_ complex, template particle picker yielded 13,181,795 particle projections. The projections were subjected to reference-free 2D classification to discard poorly defined particles, producing 641,066 particle projections for further processing. This subset of particle projections was subjected to a round of Hetero Refinement with a pixel size of 0.81 Å. A selected subset containing 498,854 projections was used as a template for another round of template picking which yielded 5,616,578 particles in Relion. These projections were subjected to the same reference-free 2D classification and maximum likelihood-based three-dimensional classification, resulting in 826,485 and 429,903 particles, respectively. The particle projections generated by the two software were combined and duplicated particles were removed, resulting in 751,101 particles, which were used to obtain the final map using a pixel size of 0.81 Å. Further 3D classification without image alignment using a mask on the receptor produced one good subset accounting for 573,877 particles, which were subsequently subjected to 3D refinement and Bayesian polishing. The final refinement generated a map with an indicated global resolution of 2.96 Å and was subsequently post-processed by DeepEMhancer^46^.

For the PrRP20–PrRPR–G_i_ complex, template particle picker and blob picker yielded 10,203,281 and 5,594,186 particle projections, respectively. The two subsets of particle projections were subjected to reference-free 2D classification to discard poorly defined particles, producing 243,879 and 240,321 particle projections for further processing, respectively. These particle projections were combined and duplicated particles were removed, resulting in 401,694 particles in CryoSPARC. Further 3D classification without image alignment using a mask including only the receptor produced one good subset, which was subsequently subjected to 3D refinement, CTF refinement, and Bayesian polishing in Relion. The final refinement generated a map with an indicated global resolution of 2.97 Å and was subsequently post-processed by DeepEMhancer.

### Model building

The initial templates of PrRPR were derived from Alphafold2^47^. Models were docked into the EM density map using UCSF Chimera^48^. The initial models were then subjected to iterative rounds of manual adjustment based on the side-chain densities of bulky aromatic amino acids in Coot^49^ and automated refinement in PHENIX^50^. The final refinement statistics were validated using the module “comprehensive validation (cryo-EM)” in PHENIX^50^. The final refinement statistics are provided in Supplementary Table S1. All structural figures were prepared using Chimera^48^, Chimera X^51^, and PyMOL (https://pymol.org/2/).

### BRET2 assays

AD293 cells (Agilent) were propagated in Dulbecco’s Modified Eagle Medium (DMEM, Thermo Fisher Scientific), enriched with 10% fetal bovine serum (FBS, Thermo Fisher Scientific), and maintained at 37 °C in a 5% CO_2_ environment. Cells were then seeded in 6-well dishes at a density of 130,000–150,000 cells per mL and allowed to incubate overnight. On the next day, cells were transfected with plasmids at a 1:1:1:1 ratio for receptor:Gα-RLuc8:Gβ:Gγ-GFP2^52^. PEI MAX 40 K (BIOHUB) was utilized to form complex with the plasmids at a ratio of 3 µL PEI per µg of plasmids, in OptiMEM (Gibco-Thermo Fisher Scientific) at a concentration of 10 ng plasmids per µL OptiMEM. After 24 h of incubation, the culture was harvested by centrifugation with PBS. The cell suspension was then allocated into a white 384-well plate at a volume of 30 µL per well, followed by the addition of 10 µL of freshly prepared 50 µM coelenterazine 400a (Yeasen). Following a 15-min equilibration period, cells were treated with 10 µL of ligand for an additional 5 min. Plates were subsequently read in a BioTek Synergy H1 microplate reader (BioTek) equipped with 395 nm (RLuc8-coelenterazine 400a) and 510 nm (GFP2) emission filters. BRET2 ratios were calculated as the ratio of the GFP2 emission to RLuc8 emission. Data were normalized to the baseline response of the ligand.

### Receptor surface expression

The cell-surface expression levels of WT or mutant PrRPR were quantified using flow cytometry. AD293 cells were seeded at a density of 1.5 × 10^5^ per well in 12-well culture plates and allowed to grow overnight. The cells were then transfected with 1.1 μg of the PrRPR construct using PEI MAX 40 K in each well and incubated for 24 h. Following this, the cells were rinsed once with PBS and detached using 0.2% (w/v) EDTA in PBS. The cells were blocked with PBS containing 5% (w/v) BSA for 15 min at room temperature, followed by incubation with the primary anti-Flag antibody (diluted with PBS containing 5% BSA at a ratio of 1:300, ABclonal) for 1 h at room temperature. The cells were then washed three times with PBS containing 1% (w/v) BSA and incubated with the anti-mouse Alexa-488-conjugated secondary antibody (diluted at a ratio of 1:1000, ABclonal) at 4 °C in the dark for 1 h. After three additional washes, the cells were collected, and the fluorescence intensity was quantified using a Luminex flow cytometer system (Guava® easyCyte) through Luminex guavaSoft 4.5 at an excitation of 488 nm and an emission of 519 nm. Approximately 10,000 cellular events per sample were collected, and the data were normalized to the WT PrRPR. Each experiment was performed at least three times, and the data are presented as means ± SEM.

### MD simulation

The simulation systems were constructed using PrRPR–G_i_ and PrRPR–G_q_ protein complexes. Protonation states of residues were established utilizing Propka3 software^53^. Using CHARMM-GUI, these structures were embedded into a 155 Å × 155 Å POPC lipid bilayer^54^, which was subsequently enclosed by a 15 Å aqueous layer. The systems were then adjusted to a sodium chloride concentration of 0.15 mol/L and supplemented with counterions. The CHARMM36m force field was employed for both amino acids and lipids^55^. The systems underwent a comprehensive 7-step equilibration process as specified by CHARMM-GUI. For each system, three independent 200 ns production runs were conducted using pmemd.cuda in Amber20^56^ within the NPT ensemble at 303.15 K and 1 atm. Long-range electrostatic interactions were handled using the Particle Mesh Ewald method, and short-range electrostatic and van der Waals interactions were managed using a 12 Å cutoff, with a gradual transition between 10 Å and 12 Å. Hydrogen bonds were constrained using SHAKE, allowing for a timestep of 2 fs. The binding free energies of the G proteins were calculated using MMPBSA.py^57^. RMSF was determined by first aligning the PrRPR proteins using the rms command in CPPTRAJ, followed by the atomicfluct command in CPPTRAJ to calculate the RMSF of residues according to the coordinate of non-hydrogen atoms^58^.

### Statistics

All functional study data were analyzed using GraphPad Prism 8.0 (Graphpad Software Inc.) and shown as means ± SEM from at least three independent experiments in triplicate. The significance was determined by one-way ANOVA with Dunnett’s multiple comparisons test, and *P < 0.05 was considered statistically significant.

## Supplementary information

Supplementary Information

## Data Availability

The atomic coordinates and the electron microscopy maps have been deposited in the Protein Data Bank (PDB) and the Electron Microscopy Data Back (EMDB) under accession numbers: 8ZPT and EMD-60354 for the PrRP20–PrRPR–G_q_ complex, 8ZPS and EMD-60353 for the PrRP20–PrRPR–G_i_ complex.
