# Supplementary material for: Molecular mechanism of prolactin-releasing peptide recognition and signaling via its G protein-coupled receptor
Source: Cell Discov. 2024 Sep 3;10:91. doi: 10.1038/s41421-024-00724-6 (PMC11369081; doi:10.1038/s41421-024-00724-6)
Supplement: Supplementary file 1 — Supplementary Information [file 41421_2024_724_MOESM1_ESM.pdf]

Supplementary Information

Molecular mechanism of prolactin-releasing peptide recognition and signaling via its G protein coupled receptor

Yang Li[1,2#], Qingning Yuan[3#], Xinheng He[1,2], Yumu Zhang[1], Chongzhao You[1,2], Canrong Wu[1], Jingru Li[1,3], H. Eric Xu[1,2*], Li-Hua Zhao[1,2,4*]

*Correspondence: eric.xu@simm.ac.cn (H. Eric Xu); zhaolihuawendy@simm.ac.cn (Li-Hua Zhao)

[#]These authors contributed equally: Yang Li, Qingning Yuan

[1]State Key Laboratory of Drug Research, Center for Structure and Function of Drug Targets, Shanghai Institute of Materia Medica, Chinese Academy of Sciences, Shanghai, 201203, China.

[2]University of Chinese Academy of Sciences, Beijing 100049, China

[3]School of Chinese Materia Medica, Nanjing University of Chinese Medicine, Nanjing 210023, China.

[4]Translational Center for Structural Biology, Ruijin Hospital, Shanghai Jiao Tong University School of Medicine, Shanghai 200025, China

Table of contents

Supplementary Fig. S1 | Purification and cryo-EM data processing of the PrRP20-PrRPR-G_q complex.

Supplementary Fig. S2 | Purification and cryo-EM data processing of PrRP20-PrRPR-G_i complex.

Supplementary Fig. S3 | Representative cryo-EM density maps of the PrRP20-PrRPR-G_q and PrRP20-PrRPR-G_i complexes.

Supplementary Fig. S4 | Effects of mutations in the ligand-binding pocket and G protein intereface of G_q coupled PrRPR by BRET2 assay.

Supplementary Fig. S5 | Effects of mutations in the ligand-binding pocket and G protein intereface of G_i coupled PrRPR by BRET2 assay.

Supplementary Fig. S6 | Conservation of the interaction sites between PrRP and PrRP receptors in different species.

Supplementary Fig. S7| Conservation of PrRP, other RF-amide peptides, and their respective receptors.

Supplementary Fig. S8| Conservation of PrRP, RY-amide peptides, and their respective receptors.

Supplementary Fig. S9 | Comparison of the interactions between N/$Q^{6.55}$ in PrRPR and neuropeptide Y receptors with their respective ligands.

Supplementary Table S1. Cryo-EM data collection, refinement and validation statistics.

Supplementary Table S2. pEC_{50} values of PrRPR mutants in response to PrRP20.

Supplementary Table S3. Span values of PrRPR mutants in response to PrRP20

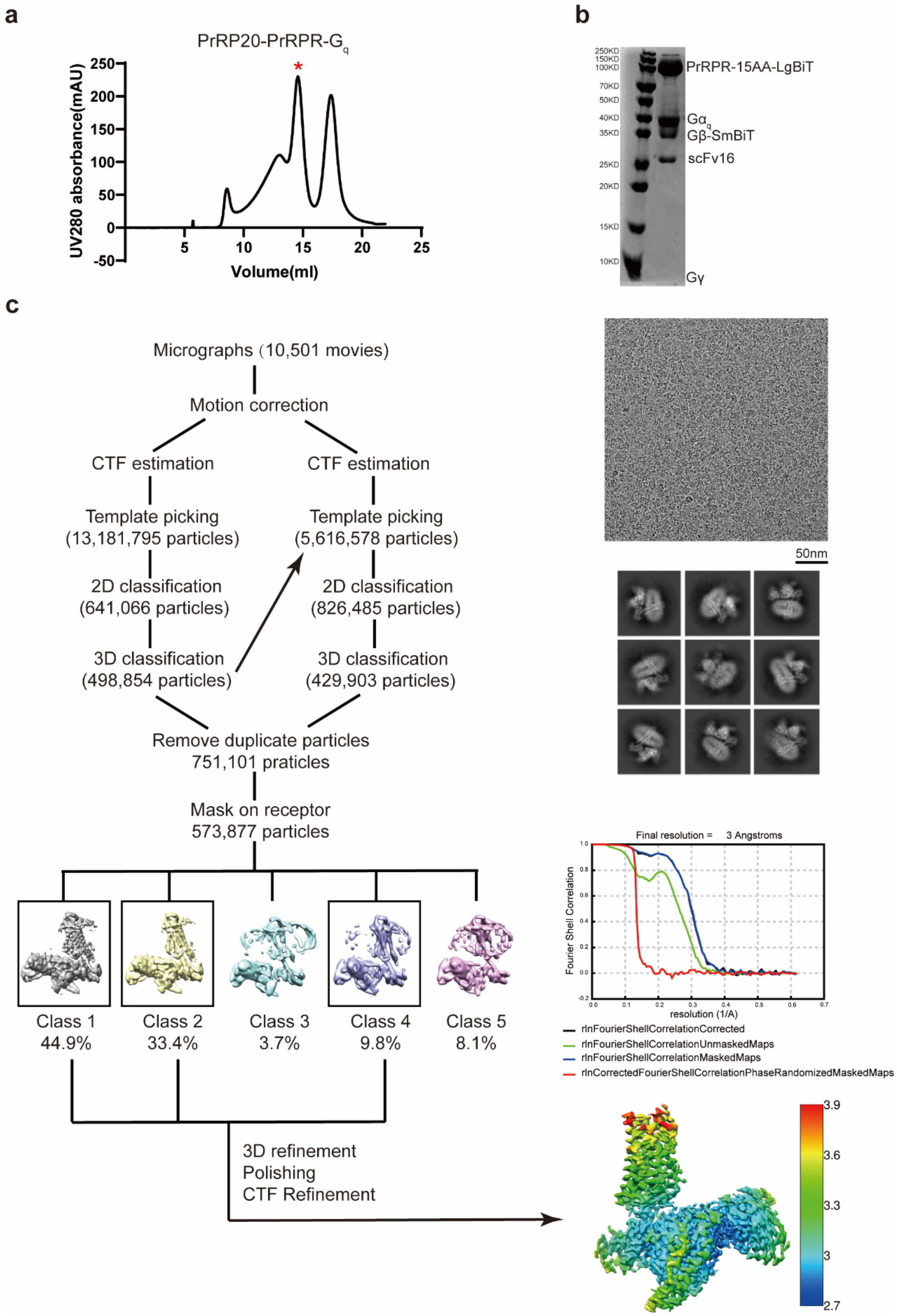

Supplementary Fig. S1 | Purification and cryo-EM data processing of the PrRP20-PrRPR-G$_q$ complex. a, b Representative size-exclusion chromatography elution profile (**a**) and SDS-PAGE analysis (**b**) of PrRP20-PrRPR-G$_q$ complex. Red asterisk

refers to complex monomer. The cryo-EM sample preparation and data collection were performed once. **c** Computational sorting of cryo-EM particle images, the “Gold-standard” FSC curve.

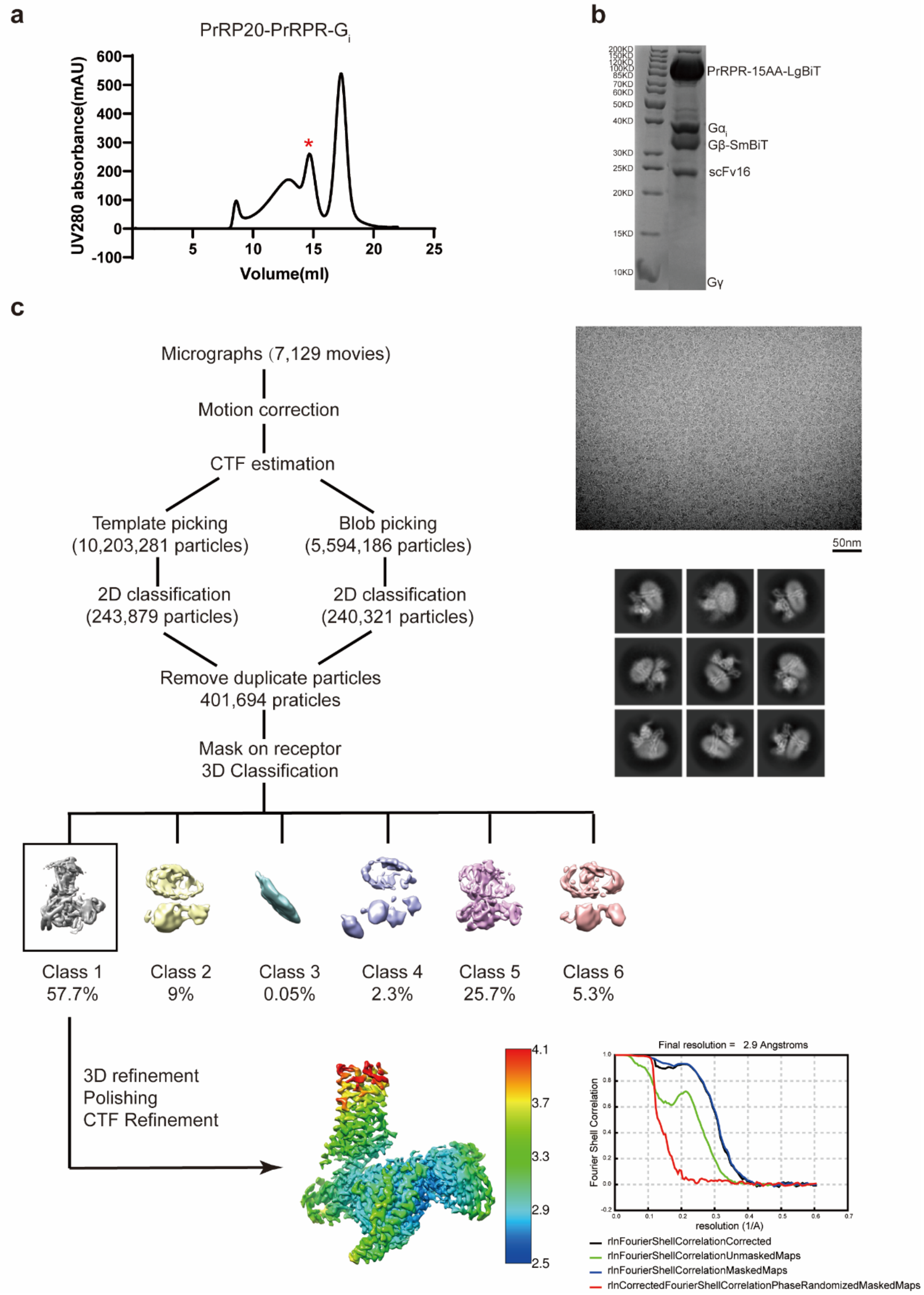

Supplementary Fig. S2 | Purification and cryo-EM data processing of PrRP20-PrRPR-G_i complex. a, b Representative size-exclusion chromatography elution profile (**a**) and SDS-PAGE analysis (**b**) of PrRP20-PrRPR-G_i complex. Red asterisk refers to complex monomer. The cryo-EM sample preparation and data collection were

performed once. **c** Computational sorting of cryo-EM particle images, the “Gold-standard” FSC curve.

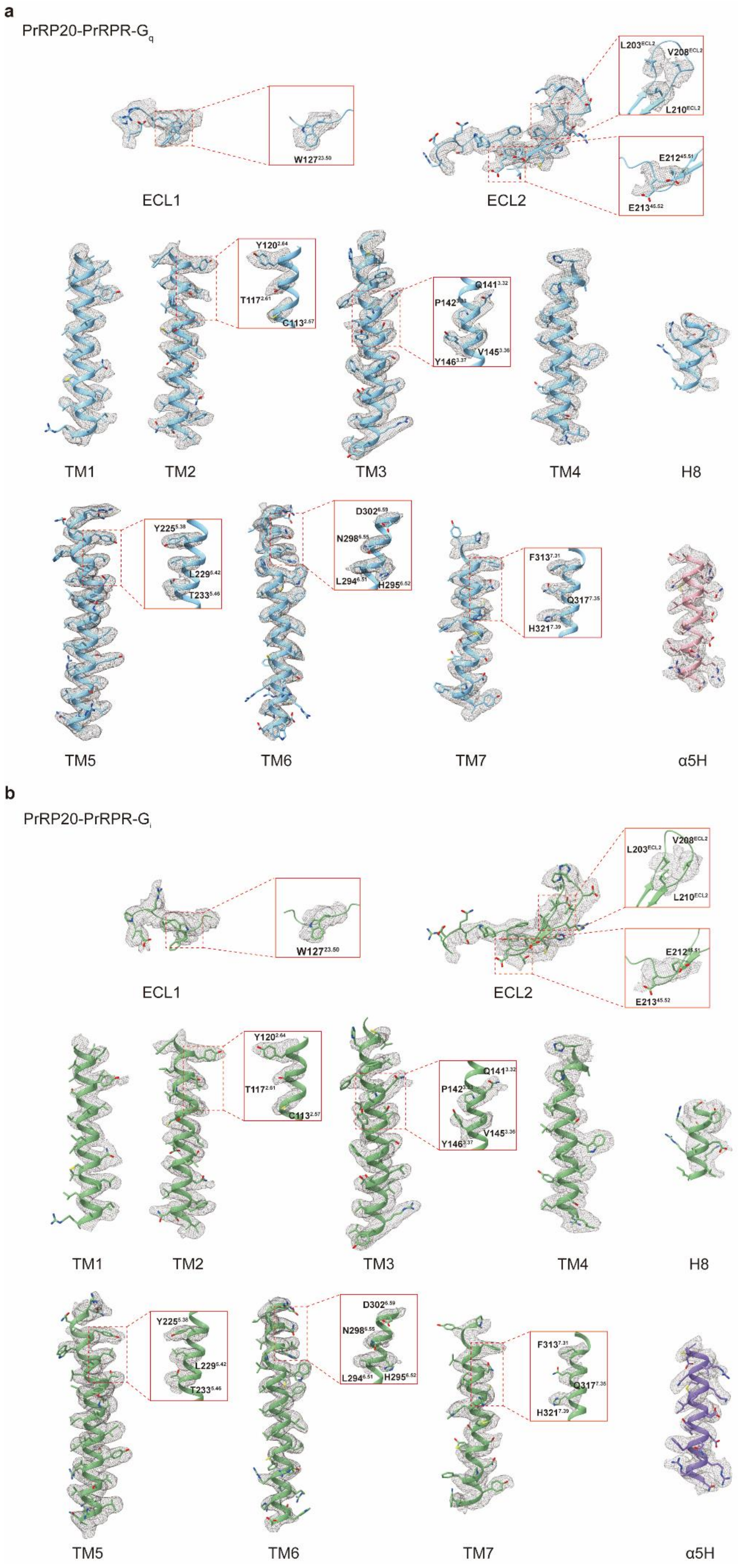

a
PrRP20-PrRPR-Gq
W127 23.50
ECL1
L203 ECL2
V208 ECL2
L210 ECL2
E212 45.51
E213 45.52
ECL2
Y120 2.64
T117 2.61
C113 2.57
Q141 3.32
P142
V145 3.36
Y146 3.37
TM1
TM2
TM3
TM4
H8
Y225 5.38
L229 5.42
T233 5.46
D302 6.59
N298 6.55
L294 6.51
H295 6.52
F313 7.31
Q317 7.35
H321 7.39
TM5
TM6
TM7
α5H
b
PrRP20-PrRPR-Gi
W127 23.50
ECL1
L203 ECL2
V208 ECL2
L210 ECL2
E212 45.51
E213 45.52
ECL2
Y120 2.64
T117 2.61
C113 2.57
Q141 3.32
P142
V145 3.36
Y146 3.37
TM1
TM2
TM3
TM4
H8
Y225 5.38
L229 5.42
T233 5.46
D302 6.59
N298 6.55
L294 6.51
H295 6.52
F313 7.31
Q317 7.35
H321 7.39
TM5
TM6
TM7
α5H

Supplementary Fig. S3 | Representative cryo-EM density maps of the PrRP20-PrRPR-G_q and PrRP20-PrRPR-G_i complexes. a, b Cryo-EM density maps of the two extracellular loops (ECL1 and ECL2) and the seven transmembrane (TM) helices with the key PrRPR residues located at the PrRP20 binging site, in addition to helix 8, and α5 helix of G protein for G_q-bound PrRPR (**a**) and G_i-bound PrRPR (**b**) were shown.

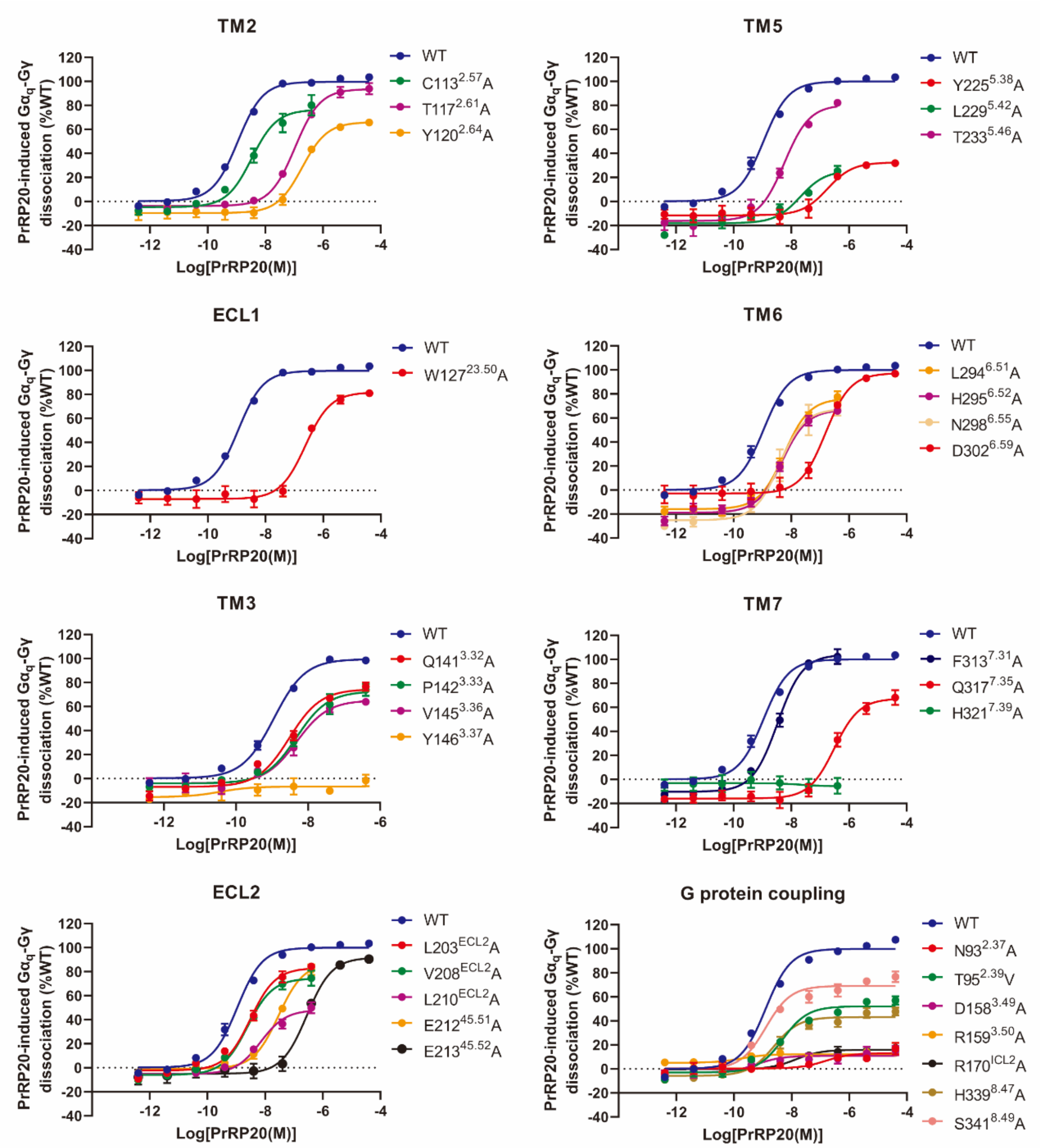

Supplementary Fig. S4 | Effects of mutations in the ligand-binding pocket and G protein intereface of G_q coupled PrRPR by BRET2 assay. AD293 cells were transfected with wild-type (WT) and PrRPR mutant constructs. BRET2 assay signals were measured after stimulation with PrRP20. Each point represents mean ± S.E.M. from three independent experiments, each consisting of triplicate measurements. The dataset links to Fig. 2i, Fig. 5i, Supplementary Table S2 and Supplementary Table S3. Source data are provided as a Source Data file.

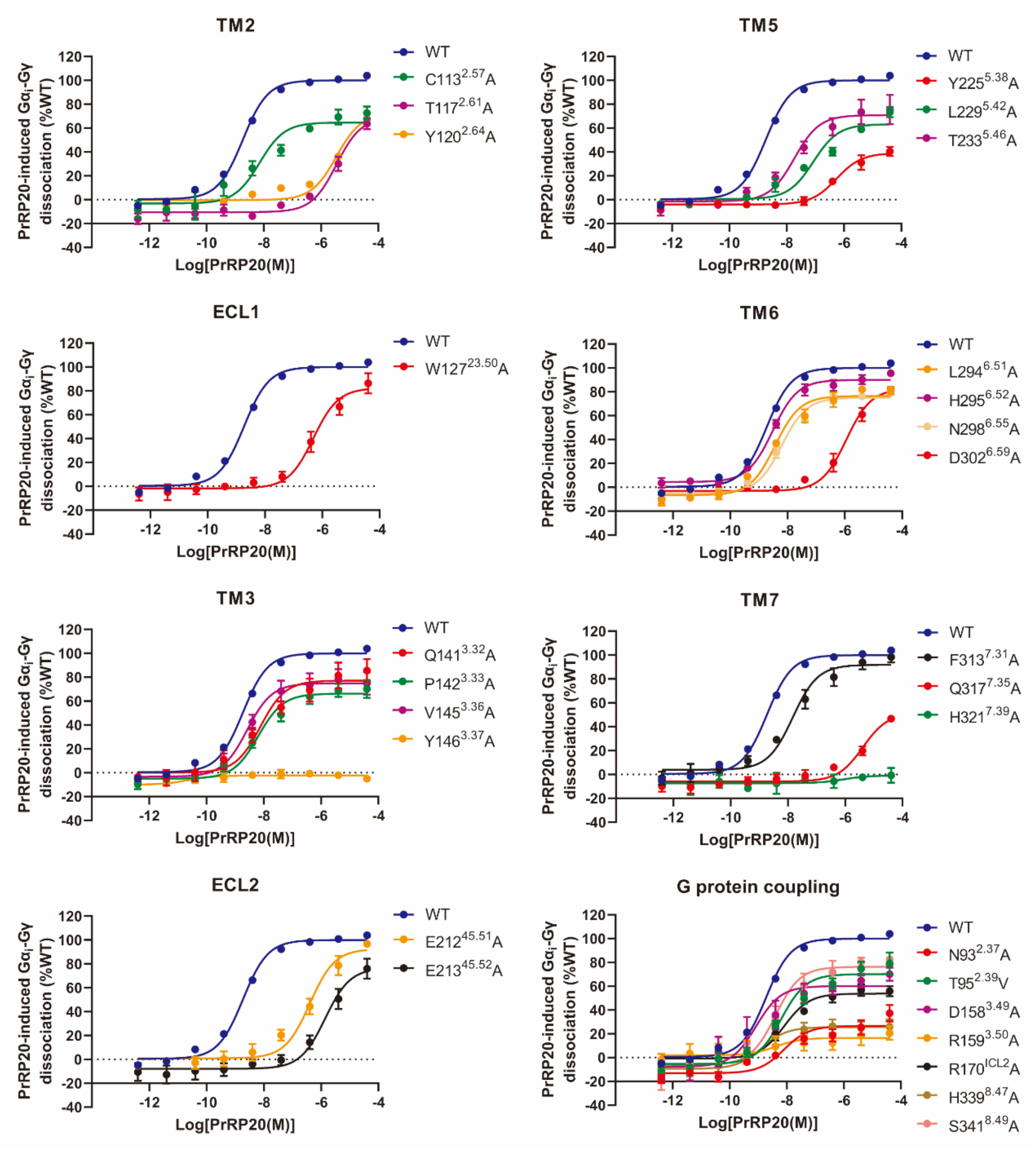

Supplementary Fig. S5 | Effects of mutations in the ligand-binding pocket and G protein intereface of G_i coupled PrRPR by BRET2 assay. Each point represents mean ± S.E.M. from three independent experiments, each consisting of triplicate measurements. The dataset links to Fig. 2j, Fig. 5j, Supplementary Table S2 and Supplementary Table S3. Source data are provided as a Source Data file.

a

b

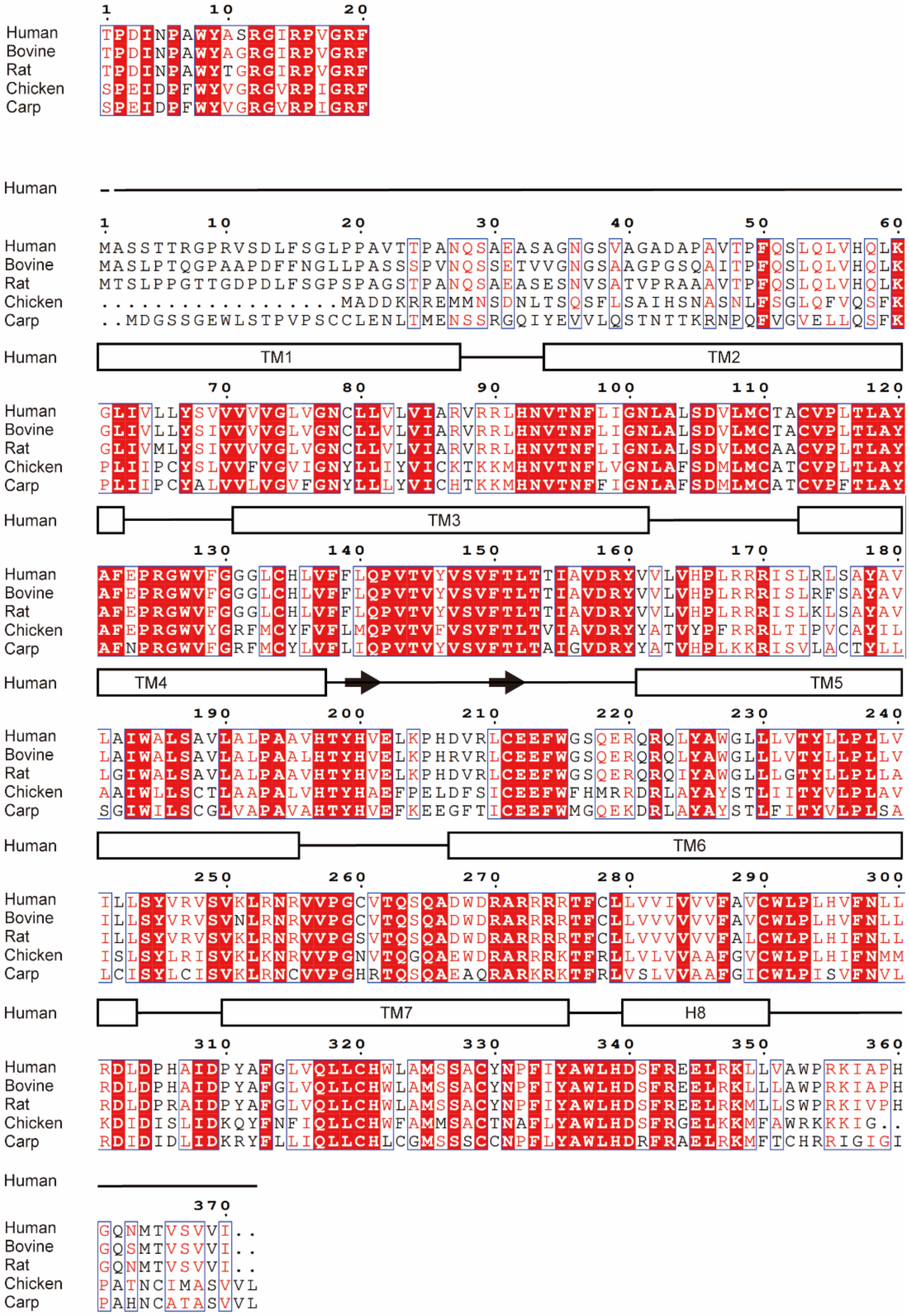

c

	2.57	2.61	2.64	23.50	3.32	3.33	3.36	3.37	ECL2	ECL2	ECL2
Human	C	T	Y	W	Q	P	V	Y	L	V	L
Bovine	C	T	Y	W	Q	P	V	Y	L	V	L
Rat	C	T	Y	W	Q	P	V	Y	L	V	L
Chicken	C	T	Y	W	Q	P	V	F	F	F	I
Carp	C	T	Y	W	Q	P	V	Y	F	F	I

	45.51	45.52	5.38	5.42	5.46	6.51	6.52	6.55	6.59	7.31	7.35	7.39
Human	E	E	Y	L	T	L	H	N	D	F	Q	H
Bovine	E	E	Y	L	T	L	H	N	D	F	Q	H
Rat	E	E	Y	L	T	L	H	N	D	F	Q	H
Chicken	E	E	Y	T	T	L	H	N	D	F	Q	H
Carp	E	E	Y	T	T	I	S	N	D	F	Q	H

Supplementary Fig. S6 | Conservation of the interaction sites between PrRP and PrRP receptors in different species. a, b Sequence alignment of the PrRP (**a**) and PrRP receptors (**b**) created by CLUSTALW (https://www.genome.jp/tools-bin/clustalw) and ESPript 3.0 (https://espript.ibcp.fr/ESPript/cgi-bin/ESPript.cgi). **c** Comparison of the conservation of residues in the binding pocket of PrRP receptors for PrRP. Residues are shown in green, magenta, light blue, and yellow, which represent polar, acidic, basic, and hydrophobic amino acids, respectively.

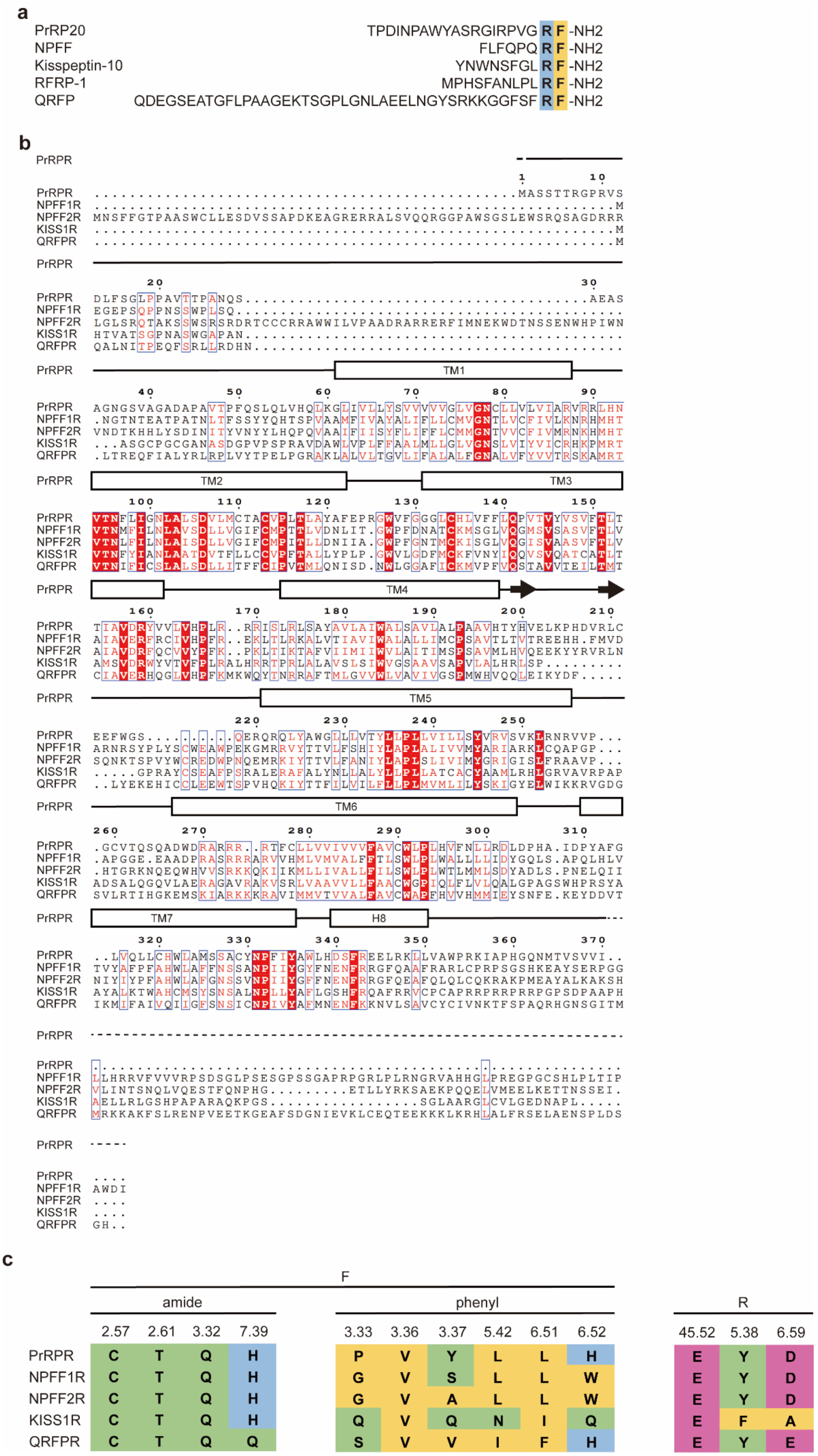
a
PrRP20 TPDINPAWYASRGIRPVG R F -NH2
NPFF FLFQPQ R F -NH2
Kisspeptin-10 YNWNSFGL R F -NH2
RFRP-1 MPHSFANLPL R F -NH2
QRFP QDEGSEATGFLPAAGEKTSGPLGNLAEELNGYSRKKGGFSF R F -NH2
b
PrRPR
NPFF1R
NPFF2R
KISS1R
QRFPR
TM1
TM2
TM3
TM4
TM5
TM6
TM7
H8
c
F
amide
phenyl
R
2.57 2.61 3.32 7.39
3.33 3.36 3.37 5.42 6.51 6.52
45.52 5.38 6.59
PrRPR C T Q H P V Y L L H E Y D
NPFF1R C T Q H G V S L L W E Y D
NPFF2R C T Q H G V A L L W E Y D
KISS1R C T Q H Q V Q N I Q E F A
QRFPR C T Q Q S V V I F H E Y E

Supplementary Fig. S7| Conservation of PrRP, other RF-amide peptides, and their respective receptors. a Sequence alignment of PrRP and other RF-amide peptides, which share a conserved C-terminal RF-amide motif. **b** Sequence alignment of the PrRPR and other RF-amide peptide receptors created by CLUSTALW and ESPript 3.0. **c** Comparison of the conservation of residues in the binding pockets of RF-amide peptide receptors for their respective ligands.

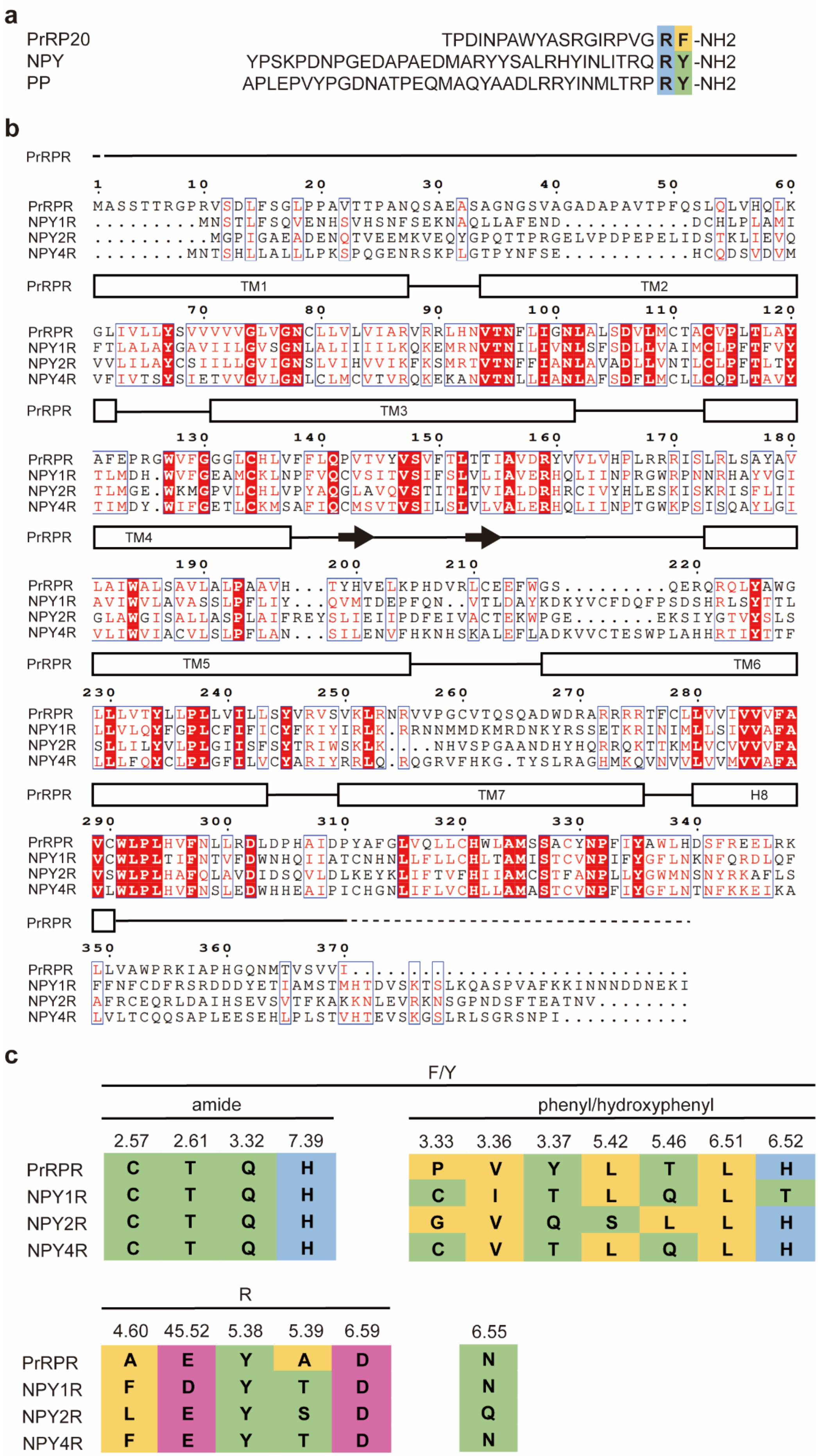
a
PrRP20 TPDINPAWYASRGIRPVGRF-NH2
NPY YPSKPDNPGEDAPAEDMARYYSALRHYINLITRQRY-NH2
PP APLEPVYPGDNATPEQMAQYAADLRRYINMLTRPRY-NH2
b
PrRPR
1 10 20 30 40 50 60
PrRPR MASSTTRGPRVSDLFSGLPPAVTTPANQSAEASAGNGSVAGADAPAVTPFQSLQLVHQLK
NPY1R .........MNSTLFSQVENHSVHSNFSEKNAQLLAFEND...........DCHLPLAMI
NPY2R ..........MGPIGAEADENQTVEEMKVEQYGPQTTPRGELVPDPEPELIDSTKLIEVQ
NPY4R ........MNTSHLLALLLPKSPQGENRSKPLGTPYNFSE...........HCQDSVDVM
PrRPR TM1 TM2
70 80 90 100 110 120
PrRPR GLIVLLYSVVVVVGLVGNCLLVLVIARVRRLHNVTNFLIGNLALSDVLMCTACVPLTLAY
NPY1R FTLALAYGAVIILGVSGNLALIIIILKQKEMRNVTNILIVNLSFSDLLVAIMCLPFTFVY
NPY2R VVLILAYCSIILLGVIGNSLVIHVVIKFKSMRTVTNFFIANLAVADLLVNTLCLPFTLTY
NPY4R VFIVTSYSIETVVGVLGNLCLMCVTVRQKEKANVTNLLIANLAFSDFLMCLLCQPLTAVY
PrRPR TM3
130 140 150 160 170 180
PrRPR AFEPRGWVFGGGLCHLVFFLQPVTVYVSVFTLTTIAVDRYVVLVHPLRRRISLRLSAYAV
NPY1R TLMDH.WVFGEAMCKLNPFVQCVSITVSIFSLVLIAVERHQLIINPRGWRPNNRHAYVGI
NPY2R TLMGE.WKMGPVLCHLVPYAQGLAVQVSTITLTVIALDRHRCIVYHLESKISKRISFLII
NPY4R TIMDY.WIFGETLCKMSAFIQCMSVTVSILSLVLVALERHQLIINPTGWKPSISQAYLGI
PrRPR TM4
190 200 210 220
PrRPR LAIWALSAVLALPAAVH...TYHVELKPHDVRLCEEFWGS.........QERQRQLYAWG
NPY1R AVIWVLAVASSLPFLIY...QVMTDEPFQN..VTLDAYKDKYVCFDQFPSDSHRLSYTTL
NPY2R GLAWGISALLASPLAIFREYSLIEIIPDFEIVACTEKWPGE.......EKSIYGTVYSLS
NPY4R VLIWVIACVLSLPFLAN...SILENVFHKNHSKALEFLADKVVCTESWPLAHHRTIYTTF
PrRPR TM5 TM6
230 240 250 260 270 280
PrRPR LLLVTYLLPLLVILLSYVRVSVKLRNRVVPGCVTQSQADWDRARRRTFCLLVVIVVVFA
NPY1R LLVLQYFGPLCFIFICYFKIYIRLK.RRNNMMDKMRDNKYRSSETKRINIMLLSIVVAFA
NPY2R SLLILYVLPLGIISFSYTRIWSKLK...NHVSPGAANDHYHQRRQKTTKMLVCVVVVFA
NPY4R LLLFQYCLPLGFILVCYARIYRRLQ.RQGRVFHKG.TYSLRAGHMKQVNVVLVVMVVAFA
PrRPR TM7 H8
290 300 310 320 330 340
PrRPR VCWLPLHVFNLLRDLDPHAIDPYAFGLVQLLCHWLAMSSACYNPFIYAWLHDSFREELRK
NPY1R VCWLPLTIFNTVFDWNHQIIATCNHNLLFLLCHLTAMISTCVNPIFYGFLNKNFQRDLQF
NPY2R VSWLPLHAFQLAVDIDSQVLDLKEYKLIFTVFHIIAMCSTFANPLLYGWMNSNYRKAFLS
NPY4R VLWLPLHVFNSLEDWHHEAIPICHGNLIFLVCHLLAMASTCVNPFIYGFLNTNFKKEIKA
PrRPR
350 360 370
PrRPR LLVAWPRKIAPHGQNMTVSVVI.............................
NPY1R FFNFCDFRSRDDDYETIAMSTMHTDVSKTSLKQASPVAFKKINNNDDNEKI
NPY2R AFRCEQRLDAIHSEVSVTFKAKKNLEVRKNSGPNDSFTEATNV........
NPY4R LVLTCQQSAPLEESEHLPLSTVHTEVSKGSLRLSGRSNPI...........
c
F/Y
amide
phenyl/hydroxyphenyl
2.57 2.61 3.32 7.39
3.33 3.36 3.37 5.42 5.46 6.51 6.52
PrRPR C T Q H P V Y L T L H
NPY1R C T Q H C I T L Q L T
NPY2R C T Q H G V Q S L L H
NPY4R C T Q H C V T L Q L H
R
4.60 45.52 5.38 5.39 6.59
6.55
PrRPR A E Y A D N
NPY1R F D Y T D N
NPY2R L E Y S D Q
NPY4R F E Y T D N

Supplementary Fig. S8| Conservation of PrRP, RY-amide peptides, and their respective receptors. a Sequence alignment of PrRP and RY-amide peptides. **b** Sequence alignment of the PrRPR and neuropeptide Y receptors created by CLUSTALW and ESPript 3.0. **c** Comparison of the conservation of residues in the binding pockets of PrRPR and neuropeptide Y receptors for their respective ligands.

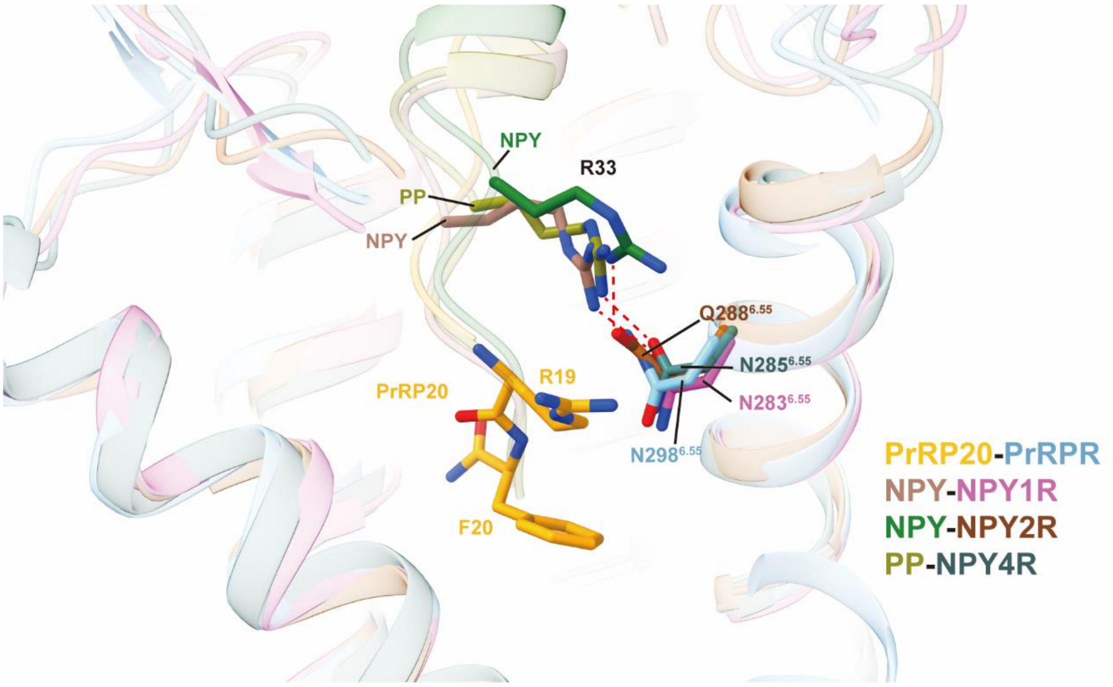

Supplementary Fig. S9 | Comparison of the interactions between N/Q$^{6.55}$ in PrRPR and neuropeptide Y receptors with their respective ligands. N283$^{6.55}$ of the NPY-NPY1R (PDB ID: 7X9A), Q288$^{6.55}$ of the NPY-NPY2R (PDB ID: 7X9B), and N285$^{6.55}$ of the PP-NPY4R (PDB ID: 7X9C) all form polar interactions with R33 of their respective ligands. In PrRPR, N298$^{6.55}$ interacts with the peptide bond between R19 and F20 in its ligand PrRP20.

Supplementary Table S1. Cryo-EM data collection, refinement and validation statistics.

	PrRP20-PrRPR-G_q	PrRP20-PrRPR-G_i
**Data collection and processing**		
Magnification	96000	105000
Voltage (kV)	300	300
Electron exposure ($e^–/Å^2$)	50	50
Defocus range (μm)	-1.2 to -1.8	-1.5 to -1.8
Pixel size (Å)	0.81	0.824
Symmetry imposed	C1	C1
Initial particle images (no.)	13,798,373	15,797,467
Final particle images (no.)	634723	436,359
Map resolution (Å)	2.96	2.97
FSC threshold	0.143	0.143
**Refinement**		
Model resolution (Å)	2.9	3.0
FSC threshold	0.143	0.143
Model-Map CC (mask)	0.75	0.76
Model composition		
Non-hydrogen atoms	9171	8835
Protein residues	1163	1135
B factors ($Å^2$)		
Protein	96.85	90.73
R.m.s. deviations		
Bond lengths (Å)	0.002	0.002
Bond angles (Å)	0.456	0.495
Validation		
MolProbity score	1.42	1.45
Clash score	7.27	7.17
Rotamer outliers (%)	0.1	0
Ramachandran plot		
Favored (%)	97.9	97.75
Allowed (%)	2.1	2.25
Disallowed (%)	0	0

Supplementary Table S2. pEC_{50} values of PrRPR mutants in response to PrRP20

BW Numbering	PrRPR Mutant	G_q-coupling		G_i-coupling		Surface expression (%WT) ± S.E.M
		BRET2 pEC_{50} ± S.E.M	*P* value (BRET2)	BRET2 pEC_{50} ± S.E.M	*P* value (BRET2)	
-	WT	8.96 ± 0.09	> 0.9999	8.73 ± 0.15	> 0.9999	100.00
2.57	C113A	8.47 ± 0.06***	0.0007	8.15 ± 0.15***	0.0008	117.89 ± 6.12
2.61	T117A	6.97 ± 0.08***	< 0.0001	5.46 ± 0.09***	< 0.0001	136.18 ± 8.57
2.64	Y120A	6.72 ± 0.05***	< 0.0001	5.47 ± 0.08***	< 0.0001	82.72 ± 4.82
23.50	W127A	6.62 ± 0.06***	< 0.0001	6.31 ± 0.09***	< 0.0001	110.71 ± 6.79
3.32	Q141A	8.52 ± 0.15**	0.0059	8.12 ± 0.14***	0.0002	129.41 ± 9.75
3.33	P142A	8.32 ± 0.03***	< 0.0001	8.19 ± 0.03**	0.0035	123.73 ± 3.32
3.36	V145A	8.32 ± 0.08***	< 0.0001	8.58 ± 0.04	0.9852	146.32 ± 3.89
3.37	Y146A	NA	NA	NA	NA	47.87 ± 9.92
ECL2	L203A	8.51 ± 0.09**	0.0021	NT	NT	138.23 ± 6.02
ECL2	V208A	8.61 ± 0.07*	0.0347	NT	NT	116.39 ± 5.10
ECL2	L210A	8.15 ± 0.04***	< 0.0001	NT	NT	102.04 ± 6.07
45.51	E212A	7.54 ± 0.02***	< 0.0001	6.40 ± 0.08***	< 0.0001	145.54 ± 12.18
45.52	E213A	6.55 ± 0.04***	< 0.0001	5.86 ± 0.14***	< 0.0001	125.64 ± 3.23
5.38	Y225A	6.75 ± 0.03***	< 0.0001	6.26 ± 0.08***	< 0.0001	75.68 ± 8.90
5.42	L229A	7.69 ± 0.20***	< 0.0001	7.07 ± 0.22***	< 0.0001	96.28 ± 1.44
5.46	T233A	8.24 ± 0.07***	< 0.0001	7.73 ± 0.17***	< 0.0001	117.44 ± 3.87
6.51	L294A	8.25 ± 0.11***	< 0.0001	8.43 ± 0.07	0.2845	124.69 ± 2.87
6.52	H295A	8.32 ± 0.05***	< 0.0001	8.52 ± 0.04	0.8032	135.06 ± 2.93
6.55	N298A	8.53 ± 0.05**	0.0042	8.20 ± 0.12**	0.0022	119.06 ± 3.00
6.59	D302A	6.82 ± 0.04***	< 0.0001	5.95 ± 0.22***	< 0.0001	154.29 ± 5.94
7.31	F313A	8.47 ± 0.15**	0.0012	7.83 ± 0.15***	< 0.0001	142.92 ± 0.50
7.35	Q317A	6.51 ± 0.07***	< 0.0001	5.38 ± 0.14***	< 0.0001	90.66 ± 10.12
7.39	H321A	NA	NA	NA	NA	38.40 ± 7.61

BRET2 assay was performed to evaluate PrRP20-induced dissociation of heterotrimeric G_q and G_i protein. Data are presented as means ± S.E.M. of three independent experiments (n=3), each consisting of triplicate measurements. All data were analyzed by two-side, one-way ANOVA with Dunnett's test. *$p< 0.05$, **$p< 0.01$, ***$p< 0.001$ compared with wild-type (WT). The dataset links to Fig. 2i, j, Supplementary Fig. S4 and Supplementary Fig. S5. BW numbering, Ballesteros & Weinstein numbering, a generic GPCR residue numbering scheme; NA, no activity; NT, Not texted.

Supplementary Table S3. Span values of PrRPR mutants in response to PrRP20

BW Numbering	PrRPR Mutant	G_q-coupling		G_i-coupling		Surface expression (%WT) ± S.E.M
		BRET2 span(%WT) ± S.E.M	*P* value (BRET2)	BRET2 span(%WT) ± S.E.M	*P* value (BRET2)	
-	WT	100.00	> 0.9999	100.00	> 0.9999	100
2.37	N93A	15.58 ± 3.15***	< 0.0001	43.46 ± 4.86***	< 0.0001	60.84 ± 2.22
2.39	T95V	55.60 ± 1.99***	< 0.0001	75.59 ± 6.77**	0.0029	79.11 ± 6.25
3.49	D158A	13.91 ± 2.93***	< 0.0001	71.83 ± 2.14***	0.0008	95.70 ± 3.27
3.50	R159A	7.31 ± 1.35***	< 0.0001	15.19 ± 1.45***	< 0.0001	113.73 ± 2.20
ICL2	R170A	16.46 ± 0.92***	< 0.0001	55.14 ± 2.05***	< 0.0001	73.35 ± 3.01
8.47	H339A	49.11 ± 3.96***	< 0.0001	35.33 ± 6.78***	< 0.0001	66.92 ± 3.00
8.49	S341A	69.46 ± 3.30***	< 0.0001	83.34 ± 0.40*	0.0465	85.98 ± 1.03

BRET2 assay was performed to evaluate PrRP20-induced dissociation of heterotrimeric G_q and G_i protein. Data are presented as means ± S.E.M. of three independent experiments (n=3), each consisting of triplicate measurements. All data were analyzed by two-side, one-way ANOVA with Dunnett's test. *p< 0.05 , **p< 0.01, ***p< 0.001 compared with wild-type (WT). The dataset links to Fig. 5i, j, Supplementary Fig. S4 and Supplementary Fig. S5.
